# Complement factor D (adipsin) mediates pressure-pain hypersensitivity post destabilization of medial meniscus injury

**DOI:** 10.1186/s13075-025-03678-6

**Published:** 2025-11-26

**Authors:** Priscilla M. Tjandra, Bethany A. Andoko, Jooyoung A. Kim, Jacob G. Brockert, Andreana G. Gomez, Sonya Sar, Megha R. Aepala, Tiffany T.K. Pham, Darren Dumlao, Hope D. Welhaven, Kelsey H. Collins

**Affiliations:** 1 Department of Orthopaedic Surgery, University of California, San Francisco, CA USA; 2 Quantitative Metabolite Analysis Center, Benioff Center for Microbiome Medicine, University of California, San Francisco, USA; 3 Oral and Craniofacial Dentistry Sciences, School of Dentistry, University of California, San Francisco, USA; 4 School of Medicine, University of California, San Francisco, USA; 5 Department of Anatomy, University of California, San Francisco, USA

## Abstract

**Background:**

Osteoarthritis (OA) is the leading cause of pain worldwide. However, clinical discordance between pain and cartilage damage presents challenges in determining the mechanisms of OA pain, thus creating a need for well-controlled models that probe the separable mechanisms of structural damage and knee pain. We previously identified that deletion of complement factor D (FD) results in increased pressure-pain hyperalgesia despite cartilage protection after destabilization of the medial meniscus (DMM) surgery. However, how these discordant OA phenotypes manifest is not understood. We employed a novel targeted lipidomics approach to elucidate the role of eicosanoids in FD-mediated pain. We hypothesize that the absence of *Cfd (FD*^*−/−*^*)* will protect cartilage but cause increased pressure-pain hyperalgesia and eicosanoid dysregulation persisting throughout OA development.

**Methods:**

Male and female *FD*^*−/−*^ and wild-type (WT) mice were challenged with DMM or remained naïve at 16 weeks old. Pressure-pain hyperalgesia was measured every two weeks for 8 weeks post-DMM. A second cohort was evaluated at 2 weeks post-DMM to investigate DMM injury response. Structural damage was scored using the Modified Mankin system. Eicosanoid profiles were characterized via liquid chromatography-mass spectrometry (LC-MS) on serum and synovial fluid samples. Statistical analysis was performed with unpaired t-test or two-way ANOVA with Sidak’s posthoc test, *p* < 0.05.

**Results:**

Unlike WT mice, *FD*^*−/−*^ mice exhibited no differences in Modified Mankin scores 8 weeks post-DMM in both sexes. As expected, *FD*^*−/−*^ and WT hyperalgesia was present at 2 weeks, persisted through 8 weeks, and was not associated with knee structural changes. Despite both sexes exhibiting similar levels of hyperalgesia, eicosanoid profiles differed. Male *FD*^*−/−*^ demonstrated greater pain-driving (12-HETE, 13-HODE) and lower pain-driving (15-HETE) and pain-suppressing (14-HDHA) abundances of eicosanoids compared to WT. Paradoxically, female *FD*^*−/−*^ exhibited bi-directional differences in pain-suppressive factors (palmitoyl ethanolamide, EPA, 14-HDHA) and lower abundances of pro-inflammatory arachidonic acid compared to WT.

**Conclusion:**

The absence of *Cfd* protects cartilage but does not prevent hyperalgesia after DMM. Changes in eicosanoid profiles suggests that loss in FD drives pain acutely and creates a hyperalgesia phenotype early in response to DMM. Eicosanoid profiling is a novel tool to mechanistically determine pain drivers in osteoarthritis.

**Supplementary Information:**

The online version contains supplementary material available at 10.1186/s13075-025-03678-6.

## Introduction

Osteoarthritis (OA), a disease characterized by the loss of cartilage lining the joint, is the leading cause of pain and disability worldwide [1, 2]. Although pain is the primary driver for patients to seek care, current pain management strategies are inadequate, highlighting the need for new drug targets and models to study OA pain. However, disentangling the mechanism of OA pain from injury and disease entrenchment is challenging. For example, pain severity is not always a result of structural damage to the joint [3]. Until recently, most studies that employ preclinical models to study OA pathogenesis rely on structural characterization of the joint and often omit pain and behavioral changes. To address this gap in knowledge, we and others [4] routinely perform pain phenotyping in all OA preclinical models to understand the concordant or divergent pain and structure phenotypes that manifest with OA in mice.

Complement factor D (FD), also known as adipsin, is a serine protease that cleaves complement factor B to activate alternative complement signaling, a key pathway in the innate immune response [5, 6]. FD is primarily secreted from adipose tissue, which our lab has demonstrated is a key driver of OA pathogenesis and pain [7]. Although prior studies have determined that the loss of several complement signaling components was protective for cartilage [8–10], we have recently demonstrated that *FD*^*−/−*^ mice displayed pronounced pressure-pain hyperalgesia, despite cartilage protection with destabilization of the medial meniscus (DMM) [11]. This model provides the opportunity to decode the discordance between structure and pain reported clinically and establishes FD as a key factor regulating fat-cartilage crosstalk in OA pain. In the present study, we leveraged this model of discordant pain and structural damage phenotype to investigate and better understand the time course changes and mechanism of pressure-pain hyperalgesia post-DMM.

Clinical data indicate that there is a dimorphism in OA, such that female patients can report more severe pain for a given amount of structural damage [12]. While historically female mice were considered a poor model to study OA, recent studies demonstrate this belief may be due to more modest structural changes in cartilage damage requiring a higher sample size to observe significant differences in histological OA scoring. To this end, several studies demonstrate that female mice do indeed develop hallmarks of OA in response to DMM [13, 14] and may demonstrate more pain for the same degree of structural damage when compared to male mice, which is representative of the clinical phenotypes described previously in many women [12].

Previously, increased synovial fluid C5 levels were associated with increased complement activation in male patients in late-stage knee OA compared to female patients [15] and early complement activation has been reported to be higher in male humans and macaques [16]. In mice, lower levels of complement activity in female mice compared to male mice are thought to be due to restricted pathway components to promote inflammation through complement C5b-9 complex [17]. In this study, we leverage the *FD*^*−/−*^ model to gain mechanistic insight into the sexual dimorphisms in pain responses due to DMM. A secondary aim of this study is to leverage the discordance in structural damage and pain in *FD*^*−/−*^ mice to better understand sexually dimorphic pain responses due to DMM.

Current treatments for pain in early OA have been limited to non-steroidal anti-inflammatory drugs (NSAID) [18, 19]. However, the efficacy of these drugs can be ineffective in OA patients for long-term use [19]. NSAIDs have been established as modulators of the complement system [20] and can influence bioactive lipid mediators called eicosanoids, which has been associated with pain. A targeted lipidomics-based eicosanoid panel [21] demonstrated that alterations in the levels of several eicosanoid species played a key role in the transition from acute to chronic hypersensitivity in the K/BxN serum transfer model of rheumatoid arthritis through toll-like receptor (TLR) 4, another key regulator of innate immunity [22]. Crosstalk between TLRs and complement signaling has been demonstrated to coordinate synergistic or exaggerated immune responses [23]. Studies have also shown that eicosanoids can be deployed downstream of complement signaling to assist with clearance and the inflammatory response to pathogens [24].

To begin to elucidate the complement-mediated mechanisms that regulate pain in DMM-induced OA immediately after injury, we used a novel targeted approach in assessing eicosanoids that are modulated by NSAIDs [21, 25–27] and can be downstream of complement signaling. As systemic soluble mediators appear to drive fat-cartilage crosstalk and OA after DMM [7], we posit that *FD*^*−/−*^ mice may demonstrate alterations in pain-driving and pain-relieving through eicosanoid mediators that act through these signaling molecules to modulate pain. Leveraging this novel eicosanoid profiling approach will give mechanistic insights to systemic and local cellular mechanisms related to pain that could lead to the development of novel therapeutic targets.

The purpose of this study was to first determine the role of FD in the onset and persistence of pain in male and female mice acutely 2 weeks after DMM, and chronically after 8 weeks of DMM. We hypothesize that the absence of *FD*^*−/−*^ will protect cartilage but result in pressure-pain hyperalgesia, which will be detectable 2 weeks post-DMM. We also hypothesize that hyperalgesia due to loss of FD would be more pronounced in male mice compared to female mice. As an exploratory hypothesis, we sought to understand if eicosanoid dysregulation due to loss of FD is observed and is driving the pressure-pain hyperalgesia phenotype, potentially indicating novel fat- and lipid-derived targets that can be probed for the development of novel therapeutic strategies for OA pain.

## Methods

### Animal studies

**Fig. 1:**
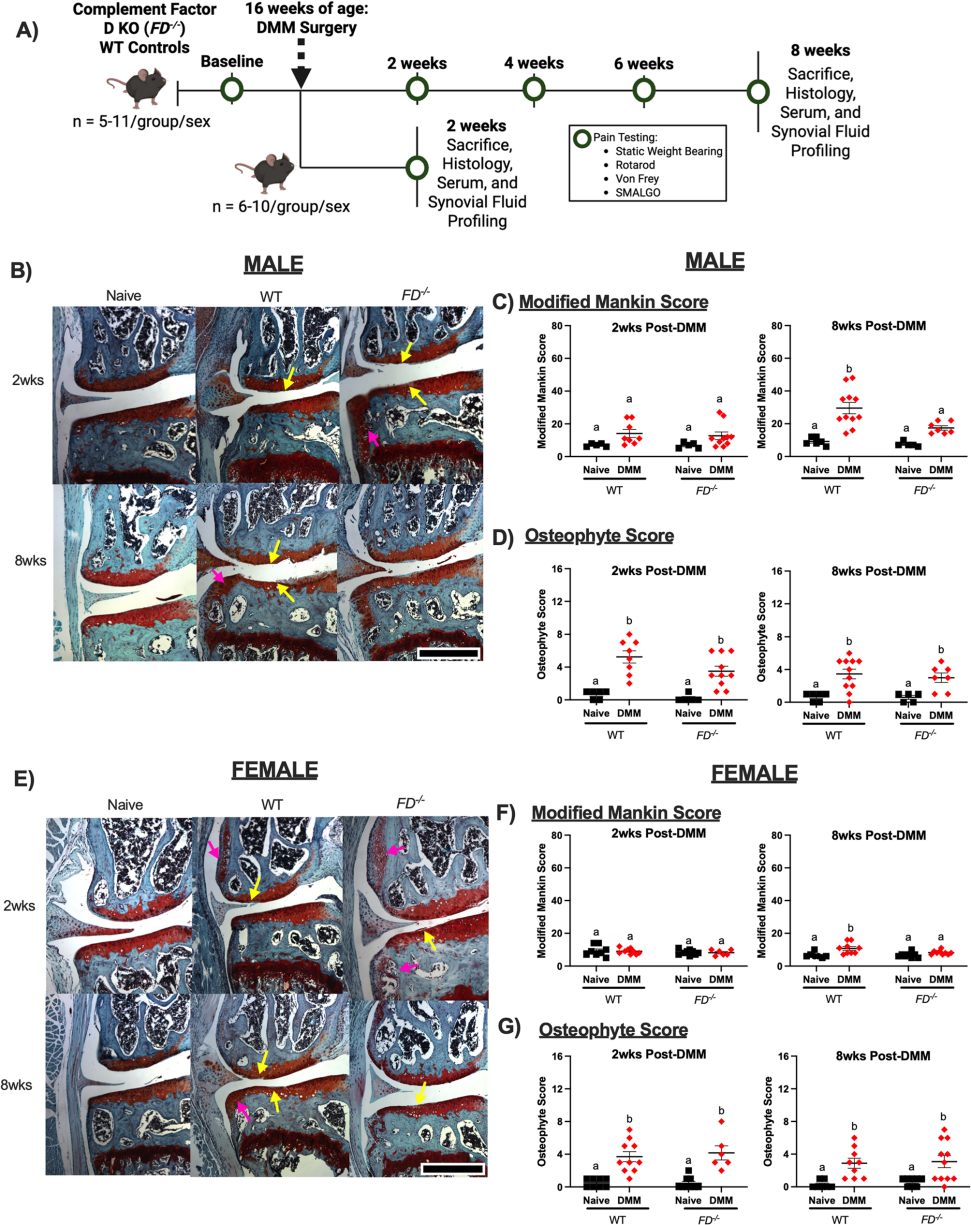
Male and female *FD*^***−/−***^ mice displayed cartilage protection. **A** Experimental design. **B**, **E** Histological slides of the medial aspect of the left knee joint from the highest scoring joints from male and female mice. Sections were stained with Safranin-O/Fast Green. Cartilage damage (yellow arrows) and osteophyte formation (pink arrows) are indicated. **C**, **F** Modified Mankin scores were greater in the WT groups compared to *FD*^*−/−*^ mice 8 weeks post-DMM in male and female mice. **D**, **G** Both sexes in both strains had greater osteophyte scores after DMM in both timepoints. Scale bar represents 500 μm. Two-way ANOVA with Sidak’s post-hoc test was used to analyze between surgery within each strain. Different letters represent *p* < 0.05 Different letters represent *p* < 0.05 when comparing naïve and DMM groups within each strain, *n* = 5–11 per surgery/sex/strain/timepoint

*FD*^*−*/−^ mice [6] (provided kindly by J. Atkinson and X. Wu; Washington University in St. Louis) were bred and maintained at the animal facility at the University of California, San Francisco. All experimental procedures were approved by the University of California, San Francisco Institutional Animal Care and Use Committee (IACUC AN199671). Male and female *FD*^*−/−*^ and wild-type (WT) (Jackson Labs #000664 ) control mice were challenged with destabilization of the medial meniscus (DMM) on the left knee joints or remained naïve at 16 weeks old (*n* = 5–11/group) as a control. Mice were sacrificed at either 2 weeks or 8 weeks post-DMM, 18 weeks or 24 weeks old in total age, at which time serum, synovial fluid, knee joints, and DRGs were collected. A timeline of these studies is presented in Fig. 1A.

### Knee joint assessment

Knee joints were prepared according to previous methods [7, 11]. In brief, joints were fixed in 4% paraformaldehyde for 24 hours and stored in 70% ethanol at 4 C°. After analyzing bone microstructure, joints were decalcified in 10% formalin solution (Cal-Ex II) for 10 hours before being processed and embedded in paraffin wax. Knee joint sections were cut at 5 μm thickness in the frontal plane and stained with Safranin-O/Fast Green or Hematoxylin and Eosin (H&E). Briefly, histological assessment was performed using the Modified Mankin, Osteophyte, and Synovitis scoring systems as previously described [7, 11]. Scores are reported as the sum of the scores given to the four quadrants of the joint in the frontal plane (medial femoral condyle, medial tibial plateau, lateral femoral condyle, and lateral tibial plateau). Analysis of cartilage and other tissues exhibiting OA pathology and osteophyte formation was done using the Modified Mankin and osteophyte scoring system respectively on joints stained with Safranin-O/Fast Green as described [7, 11]. Synovitis scores were used to grade severity of synovial pathology which is defined as synovial hyperplasia, or thickening of the lining layer, increases in cellular density in the subintimal tissues of the synovial lining, and subintimal fibrosis, or thickening of the tissues in the subintimal space of the synovial lining [7, 11, 28]. Graders were blinded to experimental groups and the average score between graders was reported for each sample. ICC scores for blinded graders was 0.94.

### Bone microstructure analysis

Whole knee joints were scanned by micro-computed tomography (SCANCO µCT50) and imaged according to the guidelines for µCT of rodent bone (energy = 55 kVP, intensity = 114 mA, 6 μm nominal voxel size, integration time = 900ms) [29] at the Skeletal Biology and Biomechanics Core at University of California, San Francisco. Analysis of the trabecular bone in the medial and lateral tibial plateau was performed by manually contouring 2D transverse slices in the region between the growth plate and the subchondral bone. Trabecular bone volume fraction (BV/TV) and bone mineral density (BMD) were determined using the manufacturer’s analysis software. Subchondral bone was assessed similarly to exclude trabecular bone in the region between the distal surface of the femoral condyles and the growth plate. Subchondral bone thickness was determined using Fiji software as previously described [30, 31].

### Immunohistochemistry to quantify sensory and sympathetic neurites

To determine the presence of and changes in neurites in the knee joint of *FD*^*−/−*^ mice compared to WT, immunohistochemistry of calcitonin gene-related peptide (CGRP) positive and tyrosine hydroxylase (TH) positive neurons was performed as described [32]. Knee joints were embedded in paraffin wax and cut serial to the sections scored for Modified Mankin in 20 μm sections at the frontal plane and region of interest was defined as similar meniscus size. Sections were blocked in 10% donkey serum and Triton-X buffer before incubation with anti-CGRP and TH primary antibodies (Bio-Rad, Millipore Sigma) overnight at 4 °C. Following 3 washes of TNT buffer, sections were incubated in Alexa Fluor™ 647 and Alexa Fluor™ 594 secondary antibodies (Jackson ImmunoResearch) for three hours at room temperature. The sections were then washed three times before incubation in DAPI (Sigma Aldrich) for 5 minutes. Three final washes were done before mounting with Invitrogen Fluoromount-G™ Mounting Medium (Fisher Scientific). Serial tiled images were taken on 10x objective images using confocal microscopy (Leica DMi8 Inverted Microscope). Tissues analyzed in neurite quantification includes the articular cartilage, subchondral bone, cruciate ligaments, meniscus, and synovium in the medial femoral, medial tibial, lateral femoral, and lateral tibial quadrants as shown in Supplementary Figs. 4 & 5. Images were processed in Fiji to exclude bone marrow, surrounding muscle, and fascia outside of the synovial lining. Contrast and brightness were adjusted to distinguish positive labels on the surface of the tissues. Positive labels were visually identified by visually comparing the brightness of Alexa Fluor™ 647 and Alexa Fluor™ 594 compared to the signal in the adjusted negative control. SNT for neuroanatomy plug-in was used to quantify positive signals by manual tracing by three graders blinded to experimental groups [30, 33]. The average number of positive CGRP and TH signal pixels was measured.

### Pain assessments and behavioral testing

Pain assessments were conducted one week before DMM and 2-, 4-, 6-, and 8-weeks post-surgery. All mice were randomized and acclimatized to the behavioral suite and equipment prior to testing. Behavioral assessments were conducted with investigators blinded to experimental groups. Pressure-pain hyperalgesia was measured using a Small Animal Algometer [7, 11] (SMALGO, Bioseb). Three to five trials of each assay for the surgical limb and nonsurgical limb were collected by applying a steady force to the lateral aspect of each limb until the mouse showed signs of discomfort such as squeaking, paw withdrawal, or grimacing. The average of these trials was reported and a maximum value of 450 g was employed to avoid tissue damage to the joint [7].

Side-to-side limb loading was measured via static incapacitance testing (Bioseb). Mice were placed in the restrainer to acclimate for ~ 2 minutes. Load bearing measurements for each limb were taken once the mouse was calm, had both feet on each of the force sensors, and had its paws placed on the ramp at the front. Incapacitance is measured as the difference between the surgical and contralateral limbs. Three to five trials were measured, and the average was reported.

To assess mechanical allodynia, a Von Frey assay was used as described [34]. Mice were placed in a box with a wire mesh bottom and left to acclimate for 20 minutes. Once acclimated, force was applied to the mid-plantar of the surgical limb paw three times using one in a series of five Von Frey filaments ranging from 0.16 g to 1.4 g. Paw withdrawal was noted as either “positive” or “negative response”. This was then repeated with each of the five Von Frey filaments, with one repeating filament for a total of 6. The order of filaments was random between observers. Paw withdrawal patterns were assessed as previously described to determine the average 50% paw withdrawal threshold from the three repetitions [34].

To assess motor coordination, mice were placed on a rotarod wheel with an initial speed of 4 rpm. Using the ramp function, the speed increased to a maximum of 40 rpm in 120 seconds. The time and the maximum speed at which the mouse fell was noted. The average of three to five trials was reported [35].

### Eicosanoid profiling by targeted lipidomics

Serum and synovial fluid [36] samples were collected and extracted from *FD*^*−/−*^ and WT DMM mice to determine systemic and local changes in eicosanoid profiles using a novel dual extraction method that separates proteins from metabolites from a single sample [11]. In brief, all samples were extracted with methanol, vortexed, and placed at −20 °C for 30 minutes to promote protein precipitation. Next, the supernatant containing metabolites was collected and dried via vacuum concentration. A 5 µL aliquot of each sample was subjected to a targeted metabolomic panel of 40 known eicosanoids, analyzed at the Quantitative Metabolite Analysis Center at the University of California, San Francisco [21] on a Shimadzu 30-AD UPLC in series with a SCIEX 7500 Triple Quadrupole Mass Spectrometer. Analytes were chromatographically separated using a Kinetex 2.6 μm Polar C18 100Å, liquid chromatography (LC) column 100 × 3.0 mm (Phenomenex, cat #00D-4759-Y0) with a mobile phase scheme of [A] water + 0.1% formic acid and [B] methanol + 0.1% formic acid. The LC method was set to a constant flow rate of 500 µL/min, and the timed linear gradient program consisted of: time = 0 min, 0.10% B, time = 0.1 min, 45% B, time = 2 min, 45% B, time = 16.5 min, 80% B, time = 16.6 min, 98% B, time = 18.5 min, 98% B, time = 18.6 min, 10% B, and time = 20.5, 10% B. Data was collected using polarity switching with the following source parameters: CUR = 40, GS1 = 60, GS2 = 70, Temp = 350 °C, ISVF = 4500 V (negative mode) and 5500 V (positive mode). Optimized multiple-reaction monitoring (MRM) pairs detailed in (Supplementary Tables 1 and 2) were used with a total cycle time of 0.937 s, dwell time of 1 ms, settling time = 15 ms, and pause time of 5.007ms. This method was based off of SCIEX’s comprehensive targeted method for lipid mediator analysis application note. Raw data was processed using a built-in SCIEX OS software package (version 2.1.6.59781) for peak picking, alignment, and quantitation. Eicosanoids detected were validated and confirmed with commercially bought standards.

### Statistical analysis

All results are reported as mean ± standard error of the mean. Results from pain and behavior assays were analyzed using two-way ANOVA with Tukey’s post-hoc test to determine differences between genotype and limb within each time point. Nonparametric Spearman’s correlations were calculated to determine correlative relationships between pressure-pain threshold or offloading of the surgical limb and osteophyte score, synovitis score, or significant eicosanoids as identified above. Lipidomic data was analyzed using an unpaired t-test on normalized abundances between WT and *FD*^*−/−*^ mice or on normalized abundances between male and female mice. All other outcomes are evaluated using two-way ANOVA with Sidak’s post hoc test to determine the effect of surgery within each strain. Heat maps and PLS-DA plots of lipidomic data were created using MetaboAnalyst. Statistical significance is defined as *p* < 0.05. Statistical analyses were performed using GraphPad Prism version 10.0.0 for Mac (GraphPad Software, Boston, Massachusetts USA, www.graphpad.com). To quantify inter-rater reliability, intraclass correlation coefficient (ICC) was calculated for all histological scores and neurite quantification amongst three graders using JASP version 0.95.0 for Mac [37].

## Results

### Male and female *FD*^***−/−***^ mice displayed cartilage protection after DMM surgery

As expected, *FD*^*−/−*^ male and female mice were protected from DMM-induced structural damage as shown in images of the highest Mankin Scores in each group (Fig. 1B and E). Concordant with previous analysis at 12-weeks post-DMM [11], male and female WT mice demonstrated significantly greater Modified Mankin scores between DMM and naïve groups at 8 weeks post-DMM (*p* < 0.01, *p* < 0.01), but not at 2 weeks. *FD*^*−/−*^ male and female mice displayed no significant differences between DMM and naïve groups in either time points (Fig. 1C and F). Despite cartilage protection in *FD*^*−/−*^ mice, significant osteophyte formation was present in both strains at both time points. WT and *FD*^*−/−*^ DMM groups of male and female mice had greater osteophyte scores compared to the naïve controls at 2 weeks (male: *p* < 0.01, *p* < 0.01; female: *p* < 0.01, *p* < 0.01) and 8 weeks (male: *p* < 0.01, *p* = 0.02; female: *p* = 0.01, *p* < 0.01) post-DMM (Fig. 1D and G).

**Fig. 2:**
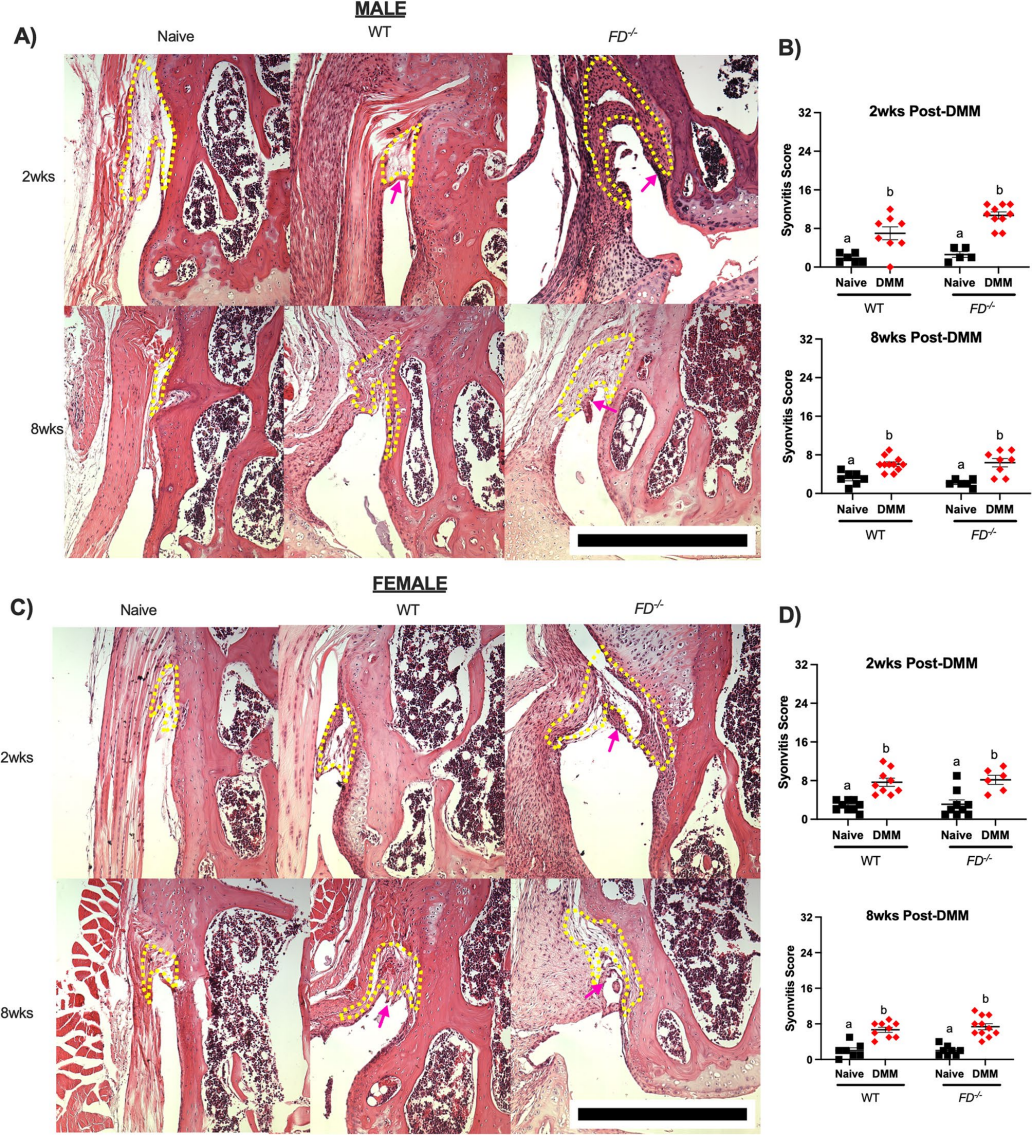
Synovitis scores increased after DMM. **A**, **C** Representative images of male and female joints stained with Hematoxylin and Eosin (H&E). WT and *FD*^*−/−*^ joints demonstrate greater presence of cells and fibrosis, or thickening, of the subintimal layer (area surrounded by yellow dotted lines) after DMM compared to naïve controls. These joints also demonstrate thickening of the synovial lining (purple arrows) when compared to naïve mice (**B**, **D**) Although all DMM groups scored higher for synovitis compared to naïve, male *FD*^*−/−*^ mice displayed greater scores than the WT DMM group at 2 weeks post-DMM. Scale bar represents 500 μm. Two-way ANOVA with Sidak’s post-hoc test was used to analyze between surgery within each strain. Different letters represent *p* < 0.05 when comparing naïve and DMM groups within each strain, *n* = 5–11 per surgery/sex/strain/timepoint

Similarly, synovitis scores were greater in all DMM groups compared to the naïve control at both 2 weeks (male: WT *p* < 0.01, *FD*^*−/−*^*p* < 0.01; female: WT *p* < 0.01, *FD*^*−/−*^*p* < 0.01) and 8 weeks (male: WT *p* < 0.01, *FD*^*−/−*^*p* < 0.01; female: WT *p* < 0.01, *FD*^*−/−*^*p* < 0.01) post-DMM (Fig. 2).

Subchondral cortical bone thickening at the distal femoral condyle was present in the medial posterior compartment in WT male mice (*p* = 0.01) at 8 weeks post-DMM. There were no differences in cortical bone thickness of the distal femoral condyles in *FD*^*−/−*^ male mice 8 weeks-post DMM. There were no other significant differences in subchondral thickness at 2 weeks post-DMM, or in female WT or *FD*^*−/−*^ mice at either time points (Supplementary Fig. 1 A). Analysis of subchondral bone changes at 2 weeks post-DMM revealed decreased BV/TV in the proximal medial tibial epiphysis in *FD*^*−/−*^ male mice (*p* = 0.02) and in the proximal lateral tibial epiphysis in *FD*^*−/−*^ female mice (*p* = 0.03), but no changes in either WT groups when compared to naïve group. There were no other differences in BV/TV of the proximal tibial epiphysis due to surgery, strain, or sex in either time points (Supplementary Fig. 1B). All subchondral bone micro-computed tomography analysis can be found in Supplementary Fig. 1.

### Pressure-pain hyperalgesia was increased in *FD*^***−/−***^ mice at 2-weeks, and persisted to 8 weeks

**Fig. 3:**
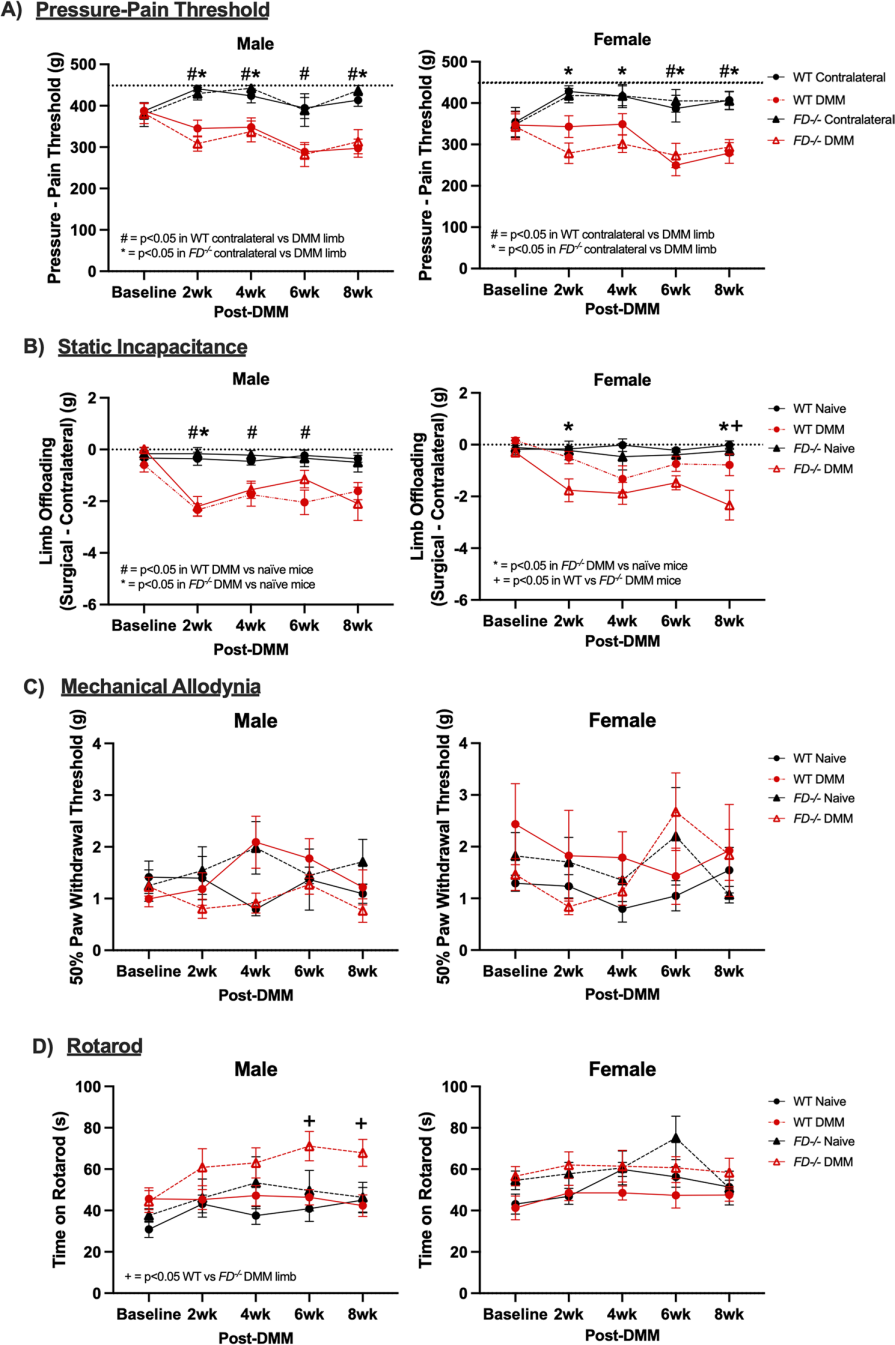
Pain phenotypes appeared at 2 weeks post-DMM and persisted through 8 weeks in both strains. **A** Male mice displayed clear pressure-pain hyperalgesia at 2 weeks and maintained for an 8-week observation period. In females, only *FD*^*−/−*^ mice exhibited hyperalgesia at early timepoints. There were no differences between *FD*^*−/−*^ and WT in either sex. **B** Offloading of the surgical limb after DMM was present in both male and female mice. **C** Mechanical allodynia and (**D**) motor coordination was no different between groups. Two-way ANOVA analysis with Tukey’s post-hoc was conducted at each timepoint with surgery and strain as main effects. # denotes *p* < 0.05 when comparing contralateral and DMM limb in WT mice, * denotes *p* < 0.05 when comparing contralateral and DMM limb in *FD*^*−/−*^ mice, + denotes *p* < 0.05 when comparing WT and *FD*^*−/−*^ DMM mice, *n* = 5–11 per surgery/sex/strain/timepoint

Pain and behavior assays were conducted one week before DMM at baseline, and 2, 4, 6, and 8 weeks after surgery. The surgical limb of WT and *FD*^*−/−*^ DMM male mice displayed decreases in pressure-pain threshold as early as 2 weeks and through 8 weeks post-DMM when compared to their contralateral limb (WT: p = < 0.01, *p* = 0.03, *p* = 0.02, *p* < 0.01; *FD*^*−/−*^: *p* < 0.01, *p* = 0.01, *p* < 0.01), but no differences when comparing between *FD*^*−/−*^ and WT DMM groups within time points. Female *FD*^*−/−*^ mice showed similar pressure-pain hyperalgesia to male *FD*^*−/−*^ and WT mice starting from 2 weeks throughout 8 weeks post-DMM (*p* < 0.01 *p* < 0.01, *p* < 0.01, *p* < 0.01). Unexpectedly, WT female mice did not demonstrate increased pain-pressure sensitivity in the surgical limb when compared to the contralateral limb until 6 and 8 weeks post-DMM (*p* = 0.03, *p* < 0.01) (Fig. 3A).

Male mice exhibited decreased loading of the DMM limb following injury in both *FD*^*−/−*^ and WT mice. However, while WT mice maintained offloading consistently through 6 weeks (*p* < 0.01, *p* < 0.05, *p* < 0.01) compared to naïve mice, *FD*^*−/−*^ mice demonstrated statistically significant increased DMM limb offloading only at 2 weeks post-DMM compared to naïve mice (*p* < 0.01) but no other time points. By 8 weeks post-DMM, there was no significant offloading in WT or *FD*^*−/−*^ male mice after DMM compared to their naïve control. Female *FD*^*−/−*^ mice exhibited significantly greater side-to-side limb offloading acutely 2 weeks after DMM and again at 8 weeks post-DMM compared to naïve mice (*p* = 0.03, *p* < 0.01), but not at 4 or 6 weeks post-DMM. In comparison, female WT mice had no differences in limb offloading between DMM and naïve mice at any time point, although female *FD*^*−/−*^ DMM mice had greater limb offloading of the DMM limb compared to WT DMM at 8 weeks post-DMM (*p* = 0.03) (Fig. 3B). There were no differences in mechanical allodynia (Fig. 3C) or motor coordination (Fig. 3D) between DMM and naïve groups, although male *FD*^*−/−*^ DMM mice demonstrated significantly longer time on the rotarod than the WT group at later timepoints (*p* = 0.02; *p* = 0.02). This is similar to what we reported at 12 weeks post DMM in this model previously [11].

To understand if the increased pain phenotypes in *FD*^*−/−*^ mice were explained by changes in histology, correlation analysis was performed between pain outcomes (pressure-pain hyperalgesia and static incapacitance) with structural changes (osteophyte and synovitis scores) to the knee between *FD*^*−/−*^ and WT mice within sex and time-point. Pressure-pain threshold and offloading of the surgical limb in *FD*^*−/−*^ mice was not significantly correlated with osteophyte scores in either sex or timepoints, although osteophyte scores were significant negatively correlated with pressure-pain threshold in WT male mice at 8 weeks post-DMM (*r* = −0.72; *p* = 0.03) and negatively correlated with static incapacitance in WT female mice at 2 weeks post-DMM (*r* = −0.66; *p* = 0.04) (Supplementary Fig. 2). Correlation analysis between synovitis and pain outcomes revealed similar conclusions. *FD*^*−/−*^ mice did not exhibit any correlations between synovitis and pressure-pain threshold or static incapacitance in either sex or time points, although there was a significant positive correlation between pressure-pain threshold and synovitis scores in female WT mice 8 weeks post-DMM (Supplementary Fig. 3).

Preliminary quantification of CGRP + and TH + neurites in the joint did not demonstrate any detectable changes between surgical groups, strain, or sex (Supplementary Figs. 4 and 5).

### Early eicosanoid changes in *FD*^***−/−***^ mice were detected in synovial fluid and serum

**Fig. 4:**
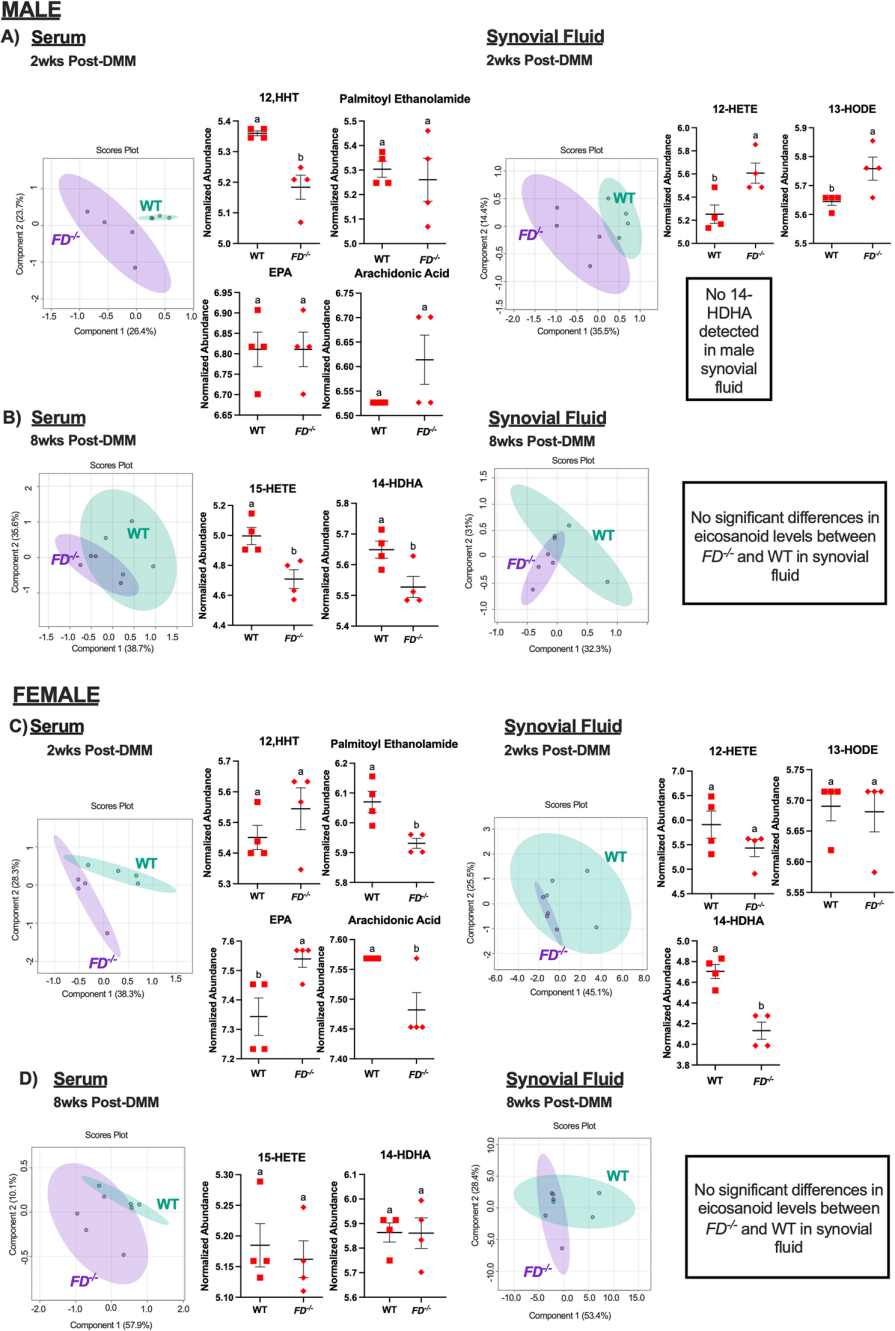
*FD*^***−/−***^ mice exhibited dysregulation of lipid profiles in the serum and synovial fluid. **A**, **C** PLS-DA plots show separation in systemic and synovial fluid lipidomic profiles of *FD*^*−/−*^ compared to WT at 2 weeks post-DMM in male and female mice. **B**, **D** At 8 weeks post-DMM, more overlap is observed in serum and synovial fluid. (**A**) At 2 weeks post-DMM, male *FD*^*-/-*^ mice displayed lower wound-healing factor (12-HHT) in the serum and higher pain driving factors (13-HODE, 12-HETE) in the synovial fluid of the surgical limb compared to WT. **B** Lower abundances in pain driving factor 15-HETE and pain-suppressive factor 14-HDHA are significantly different in the serum 8 weeks post-DMM when comparing male *FD*^*−/−*^ to WT mice. **C** Female *FD*^*−/−*^ mice demonstrated lower levels of pain suppression factor palmitoyl ethanolamide, but higher levels of pain suppression factor EPA and lower levels of pro-inflammatory factor arachidonic acid compared to WT mice. **D** At 8 weeks post-DMM, there were no significant differences in individual eicosanoid levels in female *FD*^*-/-*^ mice compared to WT mice in the serum or synovial fluid. Unpaired t-test was used to analyze between *FD*^*−/−*^ and WT mice. Different letters represent *p* < 0.05, *n* = 4 per surgery/sex/strain/timepoint

To determine the role of known eicosanoids in the *FD*^*−/−*^ pain phenotype, targeted lipidomic profiles were assessed from serum and synovial fluid of the surgical limb in DMM mice at 2 and 8 weeks post-DMM using LC-MS. PLS-DA plots from both sexes demonstrate a clear separation of lipidomic profiles at 2 weeks post-DMM in the serum and synovial fluid (Fig. 4A and B). Interestingly, the PLS-DA plot of the synovial fluid 2 weeks post-DMM from WT female mice had a higher dispersion of samples within a single cluster, indicating the samples within the WT group did not have as similar eicosanoid compositions to each other compared to the *FD*^*−/−*^ group. However, upon further investigation, we found this greater dispersion was due to the detection of all targeted eicosanoids in two female synovial fluid samples (Supplementary Table 3).

Changes in eicosanoid abundances between ^*FD*−/−^ and WT mice within synovial fluid and serum were different in male and female mice. Male *FD* ^*−/−*^ displayed a lower abundance of circulating wound healing factor 12-HHT (12-hydroxyheptadecatrienoic acid) [38] (*p* < 0.01) in the serum, as well as higher abundances of pain driving factors 12-HETE (12-hydroxyeicosatetraenoic acid) [39] and 13-HODE (13-hydroxyoctadecadienoic acid) [40] in the synovial fluid 2 weeks post-DMM compared to WT (Fig. 4A). Interestingly, in female mice, eicosanoids that are associated with pain suppression had bi-directional differences in the serum at 2 weeks post-DMM when comparing *FD*^*−/−*^ to WT mice. In the serum, the abundance of the pain suppression factor palmitoyl ethanolamide [41] was lower (*p* = 0.01), whereas the pain suppression factor EPA (eicosapentaenoic acid) [42] was higher (*p* = 0.03) in female *FD*^*-/-*^ mice when compared to WT. Additionally, the pro-inflammatory mediator arachidonic acid [43] was lower (*p *= 0.02) in the serum of female *FD*^*−/−*^ mice when compared to WT (Fig. 4C). In the synovial fluid, lipidomic assessment of *FD*^*−/−*^female mice revealed lower abundances of anti-nociceptive factor 14-HDHA (14-hydroxy-docosahexaenoic acid) [44, 45] (*p* < 0.01) compared to WT mice.

### Systemic eicosanoid changes in *FD*^***−/−***^ mice were distinct at 8-weeks post DMM in male mice

At 8 weeks, serum and synovial fluid eicosanoid profiles showed moderate overlap as shown in the PLS-DA plots in both sexes (Fig. 4B and D). *FD*^*−/−*^ male mice displayed lower abundances in 15-HETE (*p* = 0.02), a factor associated with driving pain [46], and 14-HDHA (*p* = 0.03), a factor associated with pain suppression [44, 45], in the serum 8 weeks post-DMM compared to WT mice. However, there were no eicosanoids that were significantly different between *FD*^*−/−*^ and WT mice in the synovial fluid at 8 weeks post-DMM (Fig. 4B). Female *FD*^*−/−*^ mice did not have any significant differences in individual eicosanoid levels in the serum or synovial fluid at 8 weeks post-DMM compared to WT mice (Fig. 4D).

To demonstrate the specific eicosanoid profiles in each group and over time, heat maps (Supplementary Figs. 6 & 7) and PLS-DA plots (Supplementary Fig. 8) of four-group comparisons between strain and time point are presented. To investigate correlative relationships between eicosanoids and pain, abundances of the eicosanoids mentioned above were combined and normalized across sex, strain, and timepoint and compared to pressure-pain thresholds in the synovial fluid and serum. Analysis revealed a significant positive Spearman’s correlation with normalized abundances of 14-HDHA (*r* = 0.38, *p* = 0.03) and a significant negative Spearman’s correlation with 12-HHT (*r*= −0.39, *p* = 0.03) to pressure-pain thresholds in the synovial fluid. There were no significant correlations with normalized abundances of eicosanoids of interest in the serum with pressure-pain threshold (Supplementary Table 4). Pairwise comparisons between normalized abundances of the eicosanoids mentioned above among male and female mice revealed some significant differences. Notably, abundances of pain-suppressive factors palmitoyl ethanolamide (WT: *p* < 0.01, *FD*^*−/−*^: *p* = 0.02) in the serum and 14-HDHA (WT: *p* = 0.01) in the synovial fluid 2 weeks post-DMM were higher in female mice compared to males. At 8 weeks post-DMM in the serum, 14-HDHA was lower (WT: *p* = 0.02) in females compared to males. Pain driving associated factors 12-HETE (*FD*^*−/−*^: *p* = 0.05) and 13-HODE (*FD*^*−/−*^: *p* = 0.03, WT: *p* = 0.02) were lower in abundance in the synovial fluid 2 weeks post-DMM in female mice compared to males. In the serum of female mice at 2 weeks post-DMM, wound-healing factor 12-HHT was higher in abundance (WT: *p* < 0.01) whereas arachidonic acid was lower in abundance (*FD*^*−/−*^: *p* < 0.01) compared to males (Supplementary Table 5).

## Discussion

The assumption that all pain in OA is derived from peripheral structural insults or degradation is a potential major barrier to understanding the mechanisms of OA pain. This study leverages a novel preclinical mouse model that we have recently demonstrated recapitulates the clinical reports of discordance between pain and structural damage in knee OA in both male a female mice [11]. Through constitutive knockout of *Cfd*, we establish that FD is a key mediator in driving cartilage damage in the onset of knee OA as early as 2 weeks post-DMM in male and female mice. Despite cartilage protection, *FD*^*−/−*^ animals challenged with DMM exhibited pain phenotypes that were not simply explained by histological assessments of osteophytes or synovitis in the joint. These data indicate that structural damage in the joint is not the primary driver for pain in acute and early OA in the *FD*^*−/−*^ model. Analysis of pain outcomes 2 weeks post-DMM demonstrated heightened pain sensitivity due to DMM that remained consistent throughout early onset of OA in both groups of male mice. Surprisingly, we observed that with loss of FD, male and female mice demonstrated similar pressure-pain hyperalgesia when compared to WT mice and indicates that the sensitivity to pressure-pain differs dimorphically in male and female WT mice. This suggests that FD may contribute to sexual dimorphisms in the development of pain with DMM and as OA becomes entrenched. This is supported by the sexual dimorphisms in SF and serum eicosanoid profiles between sex within genotype. Lastly, we used an innovative targeted approach to map the role of eicosanoids in synovial fluid and serum over time, which corroborates the notion that eicosanoids may be downstream mediators of FD-driven pain in OA in mice. We discovered that loss of FD in male mice resulted in increases in pain-driving (12-HETE, 13-HODE) [39, 40] and decreases in pain-suppressive (14-HDHA) [44, 45] eicosanoids, although there were also decreases in pain driving factor 15-HETE [46] after DMM when compared to WT. Interestingly, loss of FD in female mice resulted a paradoxical decrease in pain-suppressive associated factors (palmitoyl ethanolamide and 14-HDHA) [41, 44, 45] and increases in pain-suppressive factor EPA and proinflammatory factor arachidonic acid [43]. These data suggest there may be other factors, like dorsal root ganglia neurons, that may be driving the sexual dimorphism in factors driving pain. There are also likely other factors that may be contributing to and resulting from pain in this model system – but it is interesting that the same overall functional levels of hyperalgesia are observed despite the presence of different eicosanoid factors in male and female *FD*^*−/−*^ mice. Taken together, these eicosanoids may be novel targets for the development of drugs to target OA pain.

Here, we demonstrate that FD drives cartilage changes in OA in male and female mice. Congruent with existing literature, *FD*^*−/−*^ mice were protected from cartilage damage compared to WT after DMM. Recent studies, including our own, indicate that male *FD*^*−/−*^ mice showed significantly less cartilage damage in spontaneous and DMM models of established OA with similar levels of pressure-pain hyperalgesia 12 weeks post-DMM [9–11]. However, little is known about the FD and alternative complement signaling in female mice. Because female mice have been reported to display more modest cartilage damage after DMM [47], they are often excluded from OA preclinical studies. Our present data provide evidence to the contrary. With sufficient sample sizes, WT female mice displayed statistically significant cartilage damage that was not present in the *FD*^*−/−*^ group, indicating that the chondroprotective effect of in the loss of FD is not dependent on sex. These data are important for two key reasons. First, this supports the notion that female mice challenged with DMM is an intriguing model of OA pain. Second, our findings indicate that there may be sexual dimorphisms in the mechanism of FD-mediated pain-structure discordance that should be considered. WT female mice demonstrated more pain for a given amount of cartilage damage, which is concordant with reports of increased pain severity in female patients [12] and supports the notion that holistically phenotyping preclinical mouse studies with pain and behavior may provide more translationally relevant targets and mechanistic understanding.

Our findings illustrate that early pressure-pain hyperalgesia and static incapacitance are detectable acutely and are the result of DMM injury. Consistent with previous studies [48], pressure-pain hyperalgesia thresholds for both WT and *FD*^*−/−*^ mice significantly decrease at two weeks in male mice and remained consistent through the 8-week observation period, congruent with our previous work [11]. To our surprise, female *FD*^*−/−*^ mice, but not female WT, demonstrated a similar decrease as males in pressure-pain threshold at 2 weeks that was maintained through 8 weeks post-DMM when compared to the contralateral limb. While we did not observe changes in mechanical allodynia in this study due to DMM or loss of FD, other studies have reported that allodynia thresholds were lowest at 2–8 weeks post-DMM before a reversal of sensitivity after 8 weeks [14, 49]. Taken together, these findings indicate comparable pain response between *FD*^*−/−*^ male and female mice despite female mice exhibiting less severe cartilage damage, and as such, we reject the initial hypothesis that *FD*^*−/−*^ male mice would have a more profound pain phenotype compared to female mice.

While several studies demonstrate a role for FD and alternative complement signaling in driving cartilage damage, FD’s influence on pathological changes in other joint changes after DMM is unclear. This is particularly important because it is known that complement factors are produced and activated in almost all joint tissues [50], including the infrapatellar fat pad, which we recently showed strong FD expression in adiponectin-positive barcodes using spatial transcriptomics [11]. While there is a lack of studies that directly investigate the role of FD on osteophyte formation in OA, we have previously reported a robust osteophyte phenotype in *FD*^*−/−*^ mice 12 weeks post DMM [11]. Additionally, several studies have reported the absence of FD inhibits synovitis [9, 10]. In this present study, we observe synovitis, osteophytes, and subchondral bone plate sclerosis coincide with cartilage protection and is similar to previous work in fat-free lipodystrophic mice [7] and 12-week evaluation of *FD*^*−/−*^ mice [11]. These results highlight the potential separable nature of joint tissues in OA. Multiple, perhaps contradictory, roles for FD in response to DMM injury and the manifestation of pain require further investigation to understand its multiple key roles in OA progression and pathological joint tissue changes.

While osteophytes and synovitis have been implicated in pain in clinical and preclinical models [51, 52], our findings indicate that the role of FD on pain is not simply explained by semiquantitative measured changes in these outcomes by histology. Pressure-pain threshold was not significantly correlated to osteophyte or synovitis scores, suggesting that these structural changes of the knee cannot fully explain increased pain sensitivity in either sex. However, these measures are crude and many more advanced techniques and immune profiling are available. It is important to note that, by design, our work focuses on early onset OA to capture responses to injury (acute) and how FD affects the joint and pain over time (chronic). To date, most preclinical and clinical studies investigate the relationship between pain and structure during established and severe OA progression [11, 52]. Precise and focused work is needed to confirm or reject the direct role of osteophytes and synovitis in *FD*^*−/−*^ driven pain, which remains a very promising avenue for future work. Preliminary analysis of CGRP + and TH + nerve endings did not reveal any conclusive differences between strain, surgery, or sex. Future studies are needed to determine whether FD plays a role in differential sensory or sympathetic neurite sprouting after DMM. Altogether, these findings demonstrate that pain sensitivity is not simply explained by these structural changes in early-onset OA. Rather, our results suggest that pain may be regulated elsewhere in, or potentially outside of, the joint. Twelve weeks after DMM, we observed alterations in pathways associated with neutrophil trap formation, immunodeficiency, insulin signaling, and calcium signaling in bulk RNA sequencing of the dorsal root ganglia neurons that innervate the knee of *FD*^*−/−*^ vs. WT mice [11]. Our ongoing efforts aim to profile the DRGs from 2 weeks and 8 weeks post-DMM to uncover acute and chronic changes to these nerves and how they may help explain the early and lasting pain phenotype in the present *FD*^*−/−*^ male and female mice.

We have previously demonstrated that leptin and FD can drive OA pathogenesis [11]. Interestingly, eicosanoids can be triggered by leptin in the synovium [53] and contribute to the perception of pain through cytokine and COX2 activity [24, 25, 27, 53]. Moreover, eicosanoids are the targets in NSAID treatment, such as celecoxib, for OA pain and celecoxib has known sexually dimorphic responses in treatment efficacy [54]. Because NSAIDS can modulate the complement system [20], we utilized a targeted lipidomics panel to understand how eicosanoids are mediated by FD in response to injury and OA disease entrenchment. Moderate overlap of eicosanoid profiles in serum and synovial fluid for both time points suggests that eicosanoid changes occur in both serum and synovial fluid but in a time-dependent manner. As expected, PLS-DA plots demonstrate distinct clusters at 2 weeks but more overlap at 8 weeks in the serum and synovial fluid for both sexes due to FD’s role in activating the alternative pathway in response to tissue injury [55].

Differences were detected in the serum and synovial fluid at this early time point in both sexes, highlighting both the local and systemic effects of FD after DMM and further supporting the paradigm that OA is a systemic disease that affects the rest of the body, and can be affected by tissues outside the joint [7, 11]. In male mice, assessment of serum samples revealed lower circulating levels of wound-healing factor 12-HHT in *FD*^*−/−*^ mice compared to WT after DMMs. *FD*^*−/−*^ male mice exhibited higher levels of pain-driving factors 12-HETE, a molecule shown to amplify PGE2 signaling [39], and 13-HODE, an endogenous TRPV1 ligand implicated in inflammatory pain [40], in the synovial fluid compared to WT 2 weeks post-DMM. At 8 weeks post-DMM, 14-HDHA, a precursor to maresin which helps to resolve inflammatory processes through enhancing macrophage phagocytosis [44, 45], was lower in the serum in *FD*^*−/−*^ mice compared to WT. Interestingly, 15-HETE, a ligand in TRPV1 receptor activation associated with the promotion of nociceptive signalling [46], was lower in *FD*^*−/*−^ male mice in the serum 8 weeks post-DMM. Despite its role in driving pain, 15-HETE has been shown to have anti-inflammatory effects on chondrocytes and is implicated in the protection of cartilage in a monoiodacetate model of OA [56]. Our data does not support this directly as 15-HETE was lower in *FD*^*−/*−^ male mice with cartilage protection, although this may be due to the differences in the OA model used. Nevertheless, the dual and separable roles 15-HETE plays in mediating pain and cartilage damage in OA supports the premise that pain and structural damage are regulated by separable mechanisms that can be influenced by the same mediators. The shift from eicosanoid profile changes primarily in the synovial fluid at 2 weeks and primarily in the serum at 8 weeks potentially coincides with an acute to chronic pain transition in these male mice. Taken together, the combined effects of reducing inhibitory factors and additive effects of pain driving factors are concordant with the pronounced pain phenotype in *FD*^*−/−*^ male mice.

Male and female mice did not have significant changes of abundance in the same factors. Among female mice 2 weeks after DMM surgery, palmitoyl ethanolamide, an endocannabinoid-like lipid mediator with analgesic properties in patients with knee OA [41, 57], was lower in *FD*^*−/−*^ mice compared to WT in the serum. Unexpectedly however, the pain suppressing factor EPA, a molecule derived from the known anti-inflammatory mediator omega-3 [42] that has been associated with decreases in joint pain and clinical knee OA severity [58], was increased. Additionally, female *FD*^*−/−*^ mice displayed lower levels of arachidonic acid [43], compared to female WT mice in the serum, underscoring the complex, often contradictory role eicosanoids may play in response to injury. In the synovial fluid, anti-inflammatory mediator precursor 14-HDHA [44, 45] was lower in *FD*^*−/−*^ mice, which corroborates with the increased hyperalgesia to pressure-pain stimuli in *FD*^*−/−*^ mice compared to WT in Fig. 3. Unlike male mice, there were no significant differences between *FD*^*−/−*^ and WT in individual eicosanoid abundance in the serum or synovial fluid at 8 weeks, suggesting that female mice exhibit a stronger response in eicosanoids early to DMM injury compared to later timepoints in OA progression. Furthermore, sex-differences were present in the synovial fluid and serum 2 weeks post-DMM. WT, *FD*^*−/−*^, or both strains of female mice had lower abundances of pain-driving factors (12-HETE, 13-HODE) in the synovial fluid at 2 weeks post-DMM and higher levels of pain-suppression factors (palmitoyl ethanolamide, 14-HDHA) in the serum 2 weeks post-DMM when compared to males. Altogether, these findings demonstrate a sex-specific response in eicosanoid profiles to DMM at early time points and suggest that FD likely plays a bigger, sexually dimorphic role in driving pain in the acute inflammatory phase following DMM. Further investigation on this sexual dimorphism and the role eicosanoids and FD may play in sexually dimorphic pain are needed to untangle the impact of sex on OA pain after DMM.

In addition to eicosanoids, there may be other potential downstream mediators of FD that may impact OA progression. As an integral part of the complement cascade, FD drives the alternative pathway to amplify the classical and lectin pathways in the production of anaphylatoxins C3a and C5a. C3a and C5a and their receptors are key mediators of inflammation and have been implicated in driving rheumatoid joint damage [59] in addition to mechanical and inflammatory pain [60]. Although studies have determined that absence of FD results in lowered complement and anaphylatoxin levels [5, 61], it is still unclear whether they play a role in mediating structural damage and pain after DMM. Inhibition of C3a, C5a, and their receptors have been determined to reduce pain in inflammatory dysfunction [60]. However, *FD*^*−/−*^ displayed similar levels of pain to their WT control in our study, suggesting that the pain phenotypes seen may not be due to the regulation of anaphylatoxins downstream of FD. Further investigation in the role of anaphylatoxins and their receptors in FD-mediated OA is still needed.

While we and others have contributed data to support that OA can be by driven systemic factors [7, 11], it is unclear whether these systemic changes effect the complement activity in circulation or in complement components released from localized tissues. Emerging evidence have discovered that changes in intracellular complement activity, or complosome, are key drivers of disease including inflammatory-related musculoskeletal diseases, separate from circulating complement activity [62–64]. It has been theorized that this may be due to mesenchymal cells, such as synovial fibroblasts in the knee joint, which has been shown to secrete high levels of complement components [65]. Furthermore, immune cells such as monocytes, macrophages, dendritic cells, and T lymphocytes have the capability to produce complement factor D [66]. Whether these cells are able to activate a noncanonical complement response through the local release of complement factors is still unknown. The transition from circulating complement activity to complosome or vice versa may partially explain the differences in eicosanoid profiles between *FD*^*−/−*^ and WT mice at different time points post-DMM. However, further investigation is necessary to determine the role of complosome versus circulating complement activity post-DMM.

Although we did not find any significant correlations between pressure-pain threshold and the eicosanoid factors identified above, this may be due to: (1) the multiple functions FD may serve in OA pathogenesis, (2) insufficient sample size to identify if a relationship exists with these eicosanoid factors and OA outcomes, and (3) that the 40 eicosanoids measured do not completely capture the complete eicosanoid profiles of FD and WT mice after DMM. Thus, our future work will leverage untargeted lipidomic approaches to provide insight into other mediators that play a significant role in FD-associated pain and uncover novel molecular signatures to identify novel pain biomarkers and therapeutic targets for OA.

While this study provides new knowledge on the mechanisms of pain and structure mediated by FD post injury and over time in both male and female mice, it is not without limitations. First, the analysis of changes in nerve endings within the joint was preliminary. Second, a potential limitation includes the comparison of eicosanoid profiles of the serum to synovial fluid. Unlike the serum lipidome, the metabolite profile of synovial fluid may not be robust enough to comprehensively represent changes in lipidomic profiles, thus readings as false negatives. Further samples will be needed to detect smaller changes in synovial fluid. Lastly, while mouse models are valuable in disentangling mechanisms of OA pain and structure, there are immunological differences between mice and humans, specifically in alternative complement vs. classical complement signaling [67]. Future work will be needed in humanized mice to validate the role of FD driving pain as a key next step in translating these findings to the clinic and developing the eicosanoid targets.

In conclusion, this study contributes to the growing body of evidence that OA is a systemic disease that can be driven by factors outside the joint and can reciprocally affect the whole body. We demonstrate that valuable mechanistic information on OA pain can be gleaned by leveraging models that exhibit structure-pain discordance in response to DMM and by profiling this discordance over time. By global deletion of FD, we are able to recapitulate clinical pain-structure discordance, which allowed us to characterize the time course of pain and cartilage damage separately. We determined that *FD*^*−/−*^ has a chondroprotective effect and influences pressure-pain sensitivity in early onset OA in both male and female mice. Furthermore, our findings suggest that *FD*^*−/−*^ may contribute to sexual dimorphisms in pressure-pain hyperalgesia and side-to-side limb loading in WT animals. Targeted eicosanoid profiling of 40 known metabolites [21] revealed changes in pain regulation both locally and systemically. These findings demonstrate the potential of this approach to uncover key mechanistic insights into the relationship between fat, OA, and pain. Taken together, this work lays the foundation for identifying new fat-derived and bioactive lipid-mediated therapeutic targets downstream of FD that may be able to address both pain and structure in OA pathogenesis.

## Supplementary Information

Supplementary Material 1.

## Data Availability

All data needed to evaluate the conclusions in the paper are present in the article and supplementary documents.
