# Supplementary material for: Complement factor D (adipsin) mediates pressure-pain hypersensitivity post destabilization of medial meniscus injury
Source: Arthritis Res Ther. 2025 Nov 26;27:221. doi: 10.1186/s13075-025-03678-6 (PMC12659361; doi:10.1186/s13075-025-03678-6)
Supplement: Supplementary file 1 — Supplementary Material 1. [file 13075_2025_3678_MOESM1_ESM.docx]

**Supplementary Information for**

Complement factor D (adipsin) mediates pressure-pain hypersensitivity post destabilization of medial meniscus injury

Priscilla M. Tjandra^1^, Bethany A. Andoko^1^, Jooyoung A. Kim^1^, Jacob G. Brockert^4^, Andreana G. Gomez^1,2^, Sonya Sar^1^, Megha R. Aepala^1,3^, Tiffany T.K. Pham^1^, Darren Dumlao^4^, Hope D. Welhaven^1^, Kelsey H. Collins^1,5^

**Correspondence to:**

Kelsey H. Collins

Department of Orthopaedic Surgery, University of California San Francisco
San Francisco, CA 94143
kelsey.collins@ucsf.edu

**This PDF file includes:**

Supplementary Tables S1-S5
 Supplementary Figures S1-S8

**Supplemental Information**

**Supplementary Table 1. Negative Mode**

| **Compound ID** | **Q1** | **Q3** | **Edit Dwell** | **Dwell Time** | **EP** | **CE** | **CXP** | **Expected RT (min)** | **RT Tolerance (s)** |
| --- | --- | --- | --- | --- | --- | --- | --- | --- | --- |
| PGE2-d4 (355/193) | 355.2 | 193.1 | 1 | 1 | 10 | 25 | 12 | 8.1 | 30 |
| 15-deoxy-PGJ2-d4 (319/275) | 319.2 | 275.1 | 1 | 1 | 10 | 20 | 12 | 13.6 | 30 |
| 8-iso-PGF2a-d4 (357/197) | 357.2 | 197.1 | 1 | 1 | 10 | 30 | 12 | 7.2 | 30 |
| LTB4-d4 (339/197) | 339.2 | 197.1 | 1 | 1 | 10 | 22 | 12 | 12.1 | 30 |
| LXA4-d5 (356/115) | 356.2 | 115.1 | 1 | 1 | 10 | 19 | 12 | 8.9 | 30 |
| 11,12-EET-d11 (327/171) | 327.2 | 171.1 | 1 | 1 | 10 | 20 | 12 | 16.5 | 30 |
| 5-HETE-d8 (327/116) | 327.2 | 116.1 | 1 | 1 | 10 | 17 | 12 | 15.9 | 30 |
| 12-HETE-d8 (327/184) | 327.2 | 184.1 | 1 | 1 | 10 | 20 | 12 | 15.6 | 30 |
| 15-HETE-d8 (327/226) | 327.2 | 226.1 | 1 | 1 | 10 | 20 | 12 | 15.3 | 30 |
| RvE1-d4 (353/197) | 353.2 | 197.1 | 1 | 1 | 10 | 22 | 12 | 5.9 | 30 |
| RvD2-d5 (380/141) | 380.2 | 141.1 | 1 | 1 | 10 | 23 | 12 | 8.7 | 30 |
| RvD3-d5 (380/152) | 380.2 | 152.1 | 1 | 1 | 10 | 25 | 12 | 8.6 | 30 |
| Maresin 1-d5 (364/221) | 364.2 | 221.1 | 1 | 1 | 10 | 20 | 12 | 12.1 | 30 |
| 6k-PGF1a (369/163) | 369.2 | 163.1 | 1 | 1 | 10 | 35 | 12 | 6.8 | 30 |
| PGE2 and PGD2 (351/189) | 351.2 | 189.1 | 1 | 1 | 10 | 25 | 12 | 8.15 | 30 |
| PGE2 (351/175) | 351.2 | 175.1 | 1 | 1 | 10 | 25 | 12 | 8.06 | 30 |
| 15-keto-PGE2 (349/113) | 349.2 | 113.1 | 1 | 1 | 10 | 28 | 12 | 7.4 | 30 |
| 13,14-dihydro-15-keto PGE2 (351/175) | 351.2 | 175.1 | 1 | 1 | 10 | 26 | 12 | 8.1 | 30 |
| PGD2 (351/233) | 351.2 | 233.1 | 1 | 1 | 10 | 16 | 12 | 8.22 | 30 |
| 11-beta-PGF2a (353/193) | 353.2 | 193.1 | 1 | 1 | 10 | 30 | 12 | 7.5 | 30 |
| 13,14-dihydro-15-keto PGD2 (351/207) | 351.2 | 207.1 | 1 | 1 | 10 | 26 | 12 | 8.8 | 30 |
| PGJ2 (333/271) | 333.2 | 271.1 | 1 | 1 | 10 | 22 | 12 | 10.1 | 30 |
| 15-deoxy-PGJ2 (315/271) | 315.2 | 271.1 | 1 | 1 | 10 | 20 | 12 | 13.6 | 30 |
| PGF2a (353/193) | 353.2 | 193.1 | 1 | 1 | 10 | 34 | 12 | 8.55 | 30 |
| 15-keto PGF2a (351/113) | 351.2 | 113.1 | 1 | 1 | 10 | 35 | 12 | 8 | 30 |
| 13,14-dihydro-15-keto PGF2a (353/291) | 353.2 | 291.1 | 1 | 1 | 10 | 25 | 12 | 9.1 | 30 |
| 20-OH PGF2a (369/193) | 369.2 | 193 | 1 | 1 | 10 | 35 | 12 | 3.22 | 30 |
| TXB2 (369/169) | 369.2 | 169.1 | 1 | 1 | 10 | 22 | 12 | 7.7 | 30 |
| 12-HHT (279/179) | 279.2 | 179.1 | 1 | 1 | 10 | 22 | 12 | 13 | 30 |
| LTB4 (335/195) | 335.2 | 195.1 | 1 | 1 | 10 | 22 | 12 | 12.1 | 30 |
| 20-OH-LTB4 (351/195) | 351.2 | 195.1 | 1 | 1 | 10 | 24 | 12 | 6.6 | 30 |
| 20-COOH-LTB4 (365/195) | 365.1 | 195.1 | 1 | 1 | 10 | 25 | 12 | 6.16 | 30 |
| LXA4 (351/115) | 351.2 | 115.1 | 1 | 1 | 10 | 20 | 12 | 8.9 | 30 |
| 15(R)-LXA4 (351/115) | 351.2 | 115.1 | 1 | 1 | 10 | 20 | 12 | 9.1 | 30 |
| LXB4 (351/221) | 351.2 | 221.1 | 1 | 1 | 10 | 22 | 12 | 8.4 | 30 |
| 5,15-diHETE (335/115) | 335.2 | 115.1 | 1 | 1 | 10 | 22 | 12 | 11.7 | 30 |
| 5S,6R-diHETE (335/115) | 335.2 | 115.1 | 1 | 1 | 10 | 20 | 12 | 13.6 | 30 |
| 5,6 EET (319/191) | 319.2 | 191.1 | 1 | 1 | 10 | 21 | 12 | 16.7 | 30 |
| 8,9 EET (319/155) | 319.2 | 155.1 | 1 | 1 | 10 | 21 | 12 | 16.6 | 30 |
| 11,12 EET (319/167) | 319.2 | 167.1 | 1 | 1 | 10 | 21 | 12 | 16.5 | 30 |
| 14,15 EET (319/219) | 319.2 | 219.1 | 1 | 1 | 10 | 15 | 12 | 16.2 | 30 |
| 5,6 DiHETrE (337/145) | 337.2 | 145.1 | 1 | 1 | 10 | 25 | 12 | 14.4 | 30 |
| 8,9 DiHETrE (337/127) | 337.2 | 127.1 | 1 | 1 | 10 | 30 | 12 | 13.9 | 30 |
| 11,12 DiHETrE (337/167) | 337.2 | 167.1 | 1 | 1 | 10 | 25 | 12 | 13.6 | 30 |
| 14,15 DiHETrE (337/207) | 337.2 | 207.1 | 1 | 1 | 10 | 25 | 12 | 13.4 | 30 |
| 5-HETE (319/115) | 319.2 | 115.1 | 1 | 1 | 10 | 21 | 12 | 15.9 | 30 |
| 9-HETE (319/167) | 319.3 | 167.2 | 1 | 1 | 10 | 21 | 12 | 15.8 | 30 |
| 11-HETE (319/167) | 319.2 | 167.1 | 1 | 1 | 10 | 21 | 12 | 15.4 | 30 |
| 12-HETE (319/179) | 319.2 | 179.1 | 1 | 1 | 10 | 21 | 12 | 15.6 | 30 |
| 15-HETE (319/219) | 319.2 | 219.1 | 1 | 1 | 10 | 19 | 12 | 15.3 | 30 |
| 18-HETE (319/261) | 319.2 | 261.2 | 1 | 1 | 10 | 25 | 12 | 14.74 | 30 |
| 20-HETE (319/289) | 319.2 | 289.1 | 1 | 1 | 10 | 25 | 12 | 14.7 | 30 |
| 8-iso-PGF2a (353/193) | 353.2 | 193.1 | 1 | 1 | 10 | 30 | 12 | 7.2 | 30 |
| AA (303/259) | 303.2 | 259.1 | 1 | 1 | 10 | 16 | 12 | 17.8 | 30 |
| RvE1 (349/195) | 349.2 | 195.1 | 1 | 1 | 10 | 22 | 12 | 5.9 | 30 |
| RvE2 (333/199) | 333.2 | 199.1 | 1 | 1 | 10 | 22 | 12 | 10.1 | 30 |
| RvE2(333/115) | 333.2 | 115.1 | 1 | 1 | 10 | 22 | 12 | 10.1 | 30 |
| 5,15-DiHEPE (333/115) | 333.2 | 115.1 | 1 | 1 | 10 | 22 | 12 | 10.5 | 30 |
| RvE3 (333/201) | 333.2 | 201.1 | 1 | 1 | 10 | 20 | 12 | 12 | 30 |
| LXA5 (349/115) | 349.2 | 115.1 | 1 | 1 | 10 | 20 | 12 | 7.5 | 30 |
| 5-HEPE (317/115) | 317.2 | 115.1 | 1 | 1 | 10 | 19 | 12 | 14.6 | 30 |
| 11-HEPE (317/167) | 317.2 | 167.1 | 1 | 1 | 10 | 19 | 12 | 14.3 | 30 |
| 12-HEPE (317/179) | 317.2 | 179.1 | 1 | 1 | 10 | 19 | 12 | 14.4 | 30 |
| 15-HEPE (317/219) | 317.2 | 219.1 | 1 | 1 | 10 | 18 | 12 | 14.3 | 30 |
| 18-HEPE (317/259) | 317.2 | 259.1 | 1 | 1 | 10 | 16 | 12 | 14.1 | 30 |
| 8,9-EpETE (317/127) | 317.2 | 127.1 | 1 | 1 | 10 | 20 | 12 | 15.4 | 30 |
| 11,12-EpETE (317/167) | 317.2 | 167.1 | 1 | 1 | 10 | 20 | 12 | 15.4 | 30 |
| 14,15-EpETE (317/207) | 317.2 | 207.1 | 1 | 1 | 10 | 20 | 12 | 15.4 | 30 |
| 17,18-EpETE (317/215) | 317.2 | 215.1 | 1 | 1 | 10 | 20 | 12 | 15.1 | 30 |
| EPA (301/257) | 301.2 | 257.1 | 1 | 1 | 10 | 16 | 12 | 17.7 | 30 |
| RvD1 (375/141) | 375.2 | 141.1 | 1 | 1 | 10 | 21 | 12 | 9.1 | 30 |

**Supplementary Table 1. Continued**

| **Compound ID** | **Q1** | **Q3** | **Edit Dwell** | **Dwell Time** | **EP** | **CE** | **CXP** | **Expected RT (min)** | **RT Tolerance (s)** |
| --- | --- | --- | --- | --- | --- | --- | --- | --- | --- |
| 17(R)-RvD1 (375/141) | 375.2 | 141.1 | 1 | 1 | 10 | 21 | 12 | 9.3 | 30 |
| RvD2 (375/175) | 375.2 | 175.1 | 1 | 1 | 10 | 30 | 12 | 8.7 | 30 |
| RvD3 (375/147) | 375.2 | 147.1 | 1 | 1 | 10 | 25 | 12 | 8.7 | 30 |
| 17(R)-RvD3 (375/147) | 375.2 | 147.1 | 1 | 1 | 10 | 25 | 12 | 8.5 | 30 |
| RvD4 (375/101) | 375.2 | 101.1 | 1 | 1 | 10 | 22 | 12 | 10 | 30 |
| RvD5 (359/199) | 359.2 | 199.1 | 1 | 1 | 10 | 21 | 12 | 11.9 | 30 |
| PD1 (359/153) | 359.2 | 153.1 | 1 | 1 | 10 | 21 | 12 | 12.1 | 30 |
| PDX (359/153) | 359.2 | 153.1 | 1 | 1 | 10 | 21 | 12 | 11.9 | 30 |
| Maresin 1 (359/221) | 359.2 | 221.1 | 1 | 1 | 10 | 20 | 12 | 12.1 | 30 |
| Maresin 1 (359/250) | 359.2 | 250.1 | 1 | 1 | 10 | 20 | 12 | 12.1 | 30 |
| Maresin 2 (359/221) | 359.2 | 221.1 | 1 | 1 | 10 | 20 | 12 | 13.1 | 30 |
| 4-HDHA (343/101) | 343.2 | 101.1 | 1 | 1 | 10 | 17 | 12 | 16.3 | 30 |
| 7-HDHA (343/141) | 343.2 | 141.1 | 1 | 1 | 10 | 18 | 12 | 15.8 | 30 |
| 13-HDHA (343/193) | 343.2 | 193.1 | 1 | 1 | 10 | 17 | 12 | 15.6 | 30 |
| 14-HDHA (343/205) | 343.2 | 205.1 | 1 | 1 | 10 | 17 | 12 | 15.7 | 30 |
| 16-HDHA (343/233) | 343.2 | 233.1 | 1 | 1 | 10 | 20 | 12 | 15.4 | 30 |
| 17-HDHA (343/245) | 343.2 | 245.1 | 1 | 1 | 10 | 17 | 12 | 15.5 | 30 |
| 7,8-EpDPA (343/113) | 343.2 | 113.1 | 1 | 1 | 10 | 20 | 12 | 16.8 | 30 |
| 10,11-EpDPA (343/153) | 343.2 | 153.2 | 1 | 1 | 10 | 20 | 12 | 16.6 | 30 |
| 13,14-EpDPA (343/161) | 343.2 | 161.1 | 1 | 1 | 10 | 20 | 12 | 16.58 | 30 |
| 16,17-EpDPA (343/274) | 343.2 | 274.1 | 1 | 1 | 10 | 20 | 12 | 16.55 | 30 |
| 19,20-EpDPA (343/241) | 343.2 | 241.1 | 1 | 1 | 10 | 20 | 12 | 16.3 | 30 |
| DHA (327/283) | 327.2 | 283.1 | 1 | 1 | 10 | 18 | 12 | 17.8 | 30 |
| DPA (329/285) | 329.2 | 285.1 | 1 | 1 | 10 | 18 | 12 | 17.9 | 30 |
| Adrenic Acid (331/287) | 331.2 | 287.1 | 1 | 1 | 10 | 18 | 12 | 18 | 30 |
| 9-HODE (295/171) | 295.2 | 171.1 | 1 | 1 | 10 | 25 | 12 | 15 | 30 |
| 13-HODE (295/195) | 295.2 | 195.1 | 1 | 1 | 10 | 25 | 12 | 15.02 | 30 |
| 13-OxoODE (13-KODE) (293/113) | 293.2 | 113.2 | 1 | 1 | 10 | 30 | 12 | 14.84 | 30 |
| 12(13)-EpOME (295/195 | 295.3 | 195.2 | 1 | 1 | 10 | 25 | 12 | 16 | 30 |
| 9,10 DiHOME (313/201) | 313.3 | 201.3 | 1 | 1 | 10 | 30 | 12 | 13 | 30 |
| 10-Nitrolinoleate (324/277) | 324.2 | 277.1 | 1 | 1 | 10 | 18 | 12 | 16.7 | 30 |
| 9(S)HOTrE (293/171) | 293.3 | 171.1 | 1 | 1 | 10 | 25 | 12 | 13.74 | 30 |
| 13(S)HOTrE (293/195) | 293.2 | 195 | 1 | 1 | 10 | 25 | 12 | 13.94 | 30 |
| PAF (508/59) | 508.3 | 59.1 | 1 | 1 | 10 | 20 | 12 | 17.8 | 30 |
| PGE2-d4 (355/193) | 355.2 | 193.1 | 1 | 1 | 10 | 25 | 12 | 8.1 | 30 |
| 15-deoxy-PGJ2-d4 (319/275) | 319.2 | 275.1 | 1 | 1 | 10 | 20 | 12 | 13.6 | 30 |
| 8-iso-PGF2a-d4 (357/197) | 357.2 | 197.1 | 1 | 1 | 10 | 30 | 12 | 7.2 | 30 |
| LTB4-d4 (339/197) | 339.2 | 197.1 | 1 | 1 | 10 | 22 | 12 | 12.1 | 30 |
| LXA4-d5 (356/115) | 356.2 | 115.1 | 1 | 1 | 10 | 19 | 12 | 8.9 | 30 |
| 11,12-EET-d11 (327/171) | 327.2 | 171.1 | 1 | 1 | 10 | 20 | 12 | 16.5 | 30 |
| 5-HETE-d8 (327/116) | 327.2 | 116.1 | 1 | 1 | 10 | 17 | 12 | 15.9 | 30 |
| 12-HETE-d8 (327/184) | 327.2 | 184.1 | 1 | 1 | 10 | 20 | 12 | 15.6 | 30 |
| 15-HETE-d8 (327/226) | 327.2 | 226.1 | 1 | 1 | 10 | 20 | 12 | 15.3 | 30 |
| RvE1-d4 (353/197) | 353.2 | 197.1 | 1 | 1 | 10 | 22 | 12 | 5.9 | 30 |
| RvD2-d5 (380/141) | 380.2 | 141.1 | 1 | 1 | 10 | 23 | 12 | 8.7 | 30 |
| RvD3-d5 (380/152) | 380.2 | 152.1 | 1 | 1 | 10 | 25 | 12 | 8.6 | 30 |
| Maresin 1-d5 (364/221) | 364.2 | 221.1 | 1 | 1 | 10 | 20 | 12 | 12.1 | 30 |
| 6k-PGF1a (369/163) | 369.2 | 163.1 | 1 | 1 | 10 | 35 | 12 | 6.8 | 30 |
| PGE2 and PGD2 (351/189) | 351.2 | 189.1 | 1 | 1 | 10 | 25 | 12 | 8.15 | 30 |
| PGE2 (351/175) | 351.2 | 175.1 | 1 | 1 | 10 | 25 | 12 | 8.06 | 30 |
| 15-keto-PGE2 (349/113) | 349.2 | 113.1 | 1 | 1 | 10 | 28 | 12 | 7.4 | 30 |
| 13,14-dihydro-15-keto PGE2 (351/175) | 351.2 | 175.1 | 1 | 1 | 10 | 26 | 12 | 8.1 | 30 |
| PGD2 (351/233) | 351.2 | 233.1 | 1 | 1 | 10 | 16 | 12 | 8.22 | 30 |
| 11-beta-PGF2a (353/193) | 353.2 | 193.1 | 1 | 1 | 10 | 30 | 12 | 7.5 | 30 |
| 13,14-dihydro-15-keto PGD2 (351/207) | 351.2 | 207.1 | 1 | 1 | 10 | 26 | 12 | 8.8 | 30 |
| PGJ2 (333/271) | 333.2 | 271.1 | 1 | 1 | 10 | 22 | 12 | 10.1 | 30 |
| 15-deoxy-PGJ2 (315/271) | 315.2 | 271.1 | 1 | 1 | 10 | 20 | 12 | 13.6 | 30 |
| PGF2a (353/193) | 353.2 | 193.1 | 1 | 1 | 10 | 34 | 12 | 8.55 | 30 |
| 15-keto PGF2a (351/113) | 351.2 | 113.1 | 1 | 1 | 10 | 35 | 12 | 8 | 30 |
| 13,14-dihydro-15-keto PGF2a (353/291) | 353.2 | 291.1 | 1 | 1 | 10 | 25 | 12 | 9.1 | 30 |
| 20-OH PGF2a (369/193) | 369.2 | 193 | 1 | 1 | 10 | 35 | 12 | 3.22 | 30 |
| TXB2 (369/169) | 369.2 | 169.1 | 1 | 1 | 10 | 22 | 12 | 7.7 | 30 |
| 12-HHT (279/179) | 279.2 | 179.1 | 1 | 1 | 10 | 22 | 12 | 13 | 30 |
| LTB4 (335/195) | 335.2 | 195.1 | 1 | 1 | 10 | 22 | 12 | 12.1 | 30 |
| 20-OH-LTB4 (351/195) | 351.2 | 195.1 | 1 | 1 | 10 | 24 | 12 | 6.6 | 30 |
| 20-COOH-LTB4 (365/195) | 365.1 | 195.1 | 1 | 1 | 10 | 25 | 12 | 6.16 | 30 |
| LXA4 (351/115) | 351.2 | 115.1 | 1 | 1 | 10 | 20 | 12 | 8.9 | 30 |
| 15(R)-LXA4 (351/115) | 351.2 | 115.1 | 1 | 1 | 10 | 20 | 12 | 9.1 | 30 |
| LXB4 (351/221) | 351.2 | 221.1 | 1 | 1 | 10 | 22 | 12 | 8.4 | 30 |
| 5,15-diHETE (335/115) | 335.2 | 115.1 | 1 | 1 | 10 | 22 | 12 | 11.7 | 30 |
| 5S,6R-diHETE (335/115) | 335.2 | 115.1 | 1 | 1 | 10 | 20 | 12 | 13.6 | 30 |
| 5,6 EET (319/191) | 319.2 | 191.1 | 1 | 1 | 10 | 21 | 12 | 16.7 | 30 |
| 8,9 EET (319/155) | 319.2 | 155.1 | 1 | 1 | 10 | 21 | 12 | 16.6 | 30 |
| 11,12 EET (319/167) | 319.2 | 167.1 | 1 | 1 | 10 | 21 | 12 | 16.5 | 30 |
| 14,15 EET (319/219) | 319.2 | 219.1 | 1 | 1 | 10 | 15 | 12 | 16.2 | 30 |

**Supplementary Table 1. Continued**

| **Compound ID** | **Q1** | **Q3** | **Edit Dwell** | **Dwell Time** | **EP** | **CE** | **CXP** | **Expected RT (min)** | **RT Tolerance (s)** |
| --- | --- | --- | --- | --- | --- | --- | --- | --- | --- |
| 5,6 DiHETrE (337/145) | 337.2 | 145.1 | 1 | 1 | 10 | 25 | 12 | 14.4 | 30 |
| 8,9 DiHETrE (337/127) | 337.2 | 127.1 | 1 | 1 | 10 | 30 | 12 | 13.9 | 30 |
| 11,12 DiHETrE (337/167) | 337.2 | 167.1 | 1 | 1 | 10 | 25 | 12 | 13.6 | 30 |
| 14,15 DiHETrE (337/207) | 337.2 | 207.1 | 1 | 1 | 10 | 25 | 12 | 13.4 | 30 |
| 5-HETE (319/115) | 319.2 | 115.1 | 1 | 1 | 10 | 21 | 12 | 15.9 | 30 |
| 9-HETE (319/167) | 319.3 | 167.2 | 1 | 1 | 10 | 21 | 12 | 15.8 | 30 |
| 11-HETE (319/167) | 319.2 | 167.1 | 1 | 1 | 10 | 21 | 12 | 15.4 | 30 |
| 12-HETE (319/179) | 319.2 | 179.1 | 1 | 1 | 10 | 21 | 12 | 15.6 | 30 |
| 15-HETE (319/219) | 319.2 | 219.1 | 1 | 1 | 10 | 19 | 12 | 15.3 | 30 |
| 18-HETE (319/261) | 319.2 | 261.2 | 1 | 1 | 10 | 25 | 12 | 14.74 | 30 |
| 20-HETE (319/289) | 319.2 | 289.1 | 1 | 1 | 10 | 25 | 12 | 14.7 | 30 |
| 8-iso-PGF2a (353/193) | 353.2 | 193.1 | 1 | 1 | 10 | 30 | 12 | 7.2 | 30 |
| AA (303/259) | 303.2 | 259.1 | 1 | 1 | 10 | 16 | 12 | 17.8 | 30 |
| RvE1 (349/195) | 349.2 | 195.1 | 1 | 1 | 10 | 22 | 12 | 5.9 | 30 |
| RvE2 (333/199) | 333.2 | 199.1 | 1 | 1 | 10 | 22 | 12 | 10.1 | 30 |
| RvE2(333/115) | 333.2 | 115.1 | 1 | 1 | 10 | 22 | 12 | 10.1 | 30 |
| 5,15-DiHEPE (333/115) | 333.2 | 115.1 | 1 | 1 | 10 | 22 | 12 | 10.5 | 30 |
| RvE3 (333/201) | 333.2 | 201.1 | 1 | 1 | 10 | 20 | 12 | 12 | 30 |
| LXA5 (349/115) | 349.2 | 115.1 | 1 | 1 | 10 | 20 | 12 | 7.5 | 30 |
| 5-HEPE (317/115) | 317.2 | 115.1 | 1 | 1 | 10 | 19 | 12 | 14.6 | 30 |
| 11-HEPE (317/167) | 317.2 | 167.1 | 1 | 1 | 10 | 19 | 12 | 14.3 | 30 |
| 12-HEPE (317/179) | 317.2 | 179.1 | 1 | 1 | 10 | 19 | 12 | 14.4 | 30 |
| 15-HEPE (317/219) | 317.2 | 219.1 | 1 | 1 | 10 | 18 | 12 | 14.3 | 30 |
| 18-HEPE (317/259) | 317.2 | 259.1 | 1 | 1 | 10 | 16 | 12 | 14.1 | 30 |
| 8,9-EpETE (317/127) | 317.2 | 127.1 | 1 | 1 | 10 | 20 | 12 | 15.4 | 30 |
| 11,12-EpETE (317/167) | 317.2 | 167.1 | 1 | 1 | 10 | 20 | 12 | 15.4 | 30 |
| 14,15-EpETE (317/207) | 317.2 | 207.1 | 1 | 1 | 10 | 20 | 12 | 15.4 | 30 |
| 17,18-EpETE (317/215) | 317.2 | 215.1 | 1 | 1 | 10 | 20 | 12 | 15.1 | 30 |
| EPA (301/257) | 301.2 | 257.1 | 1 | 1 | 10 | 16 | 12 | 17.7 | 30 |
| RvD1 (375/141) | 375.2 | 141.1 | 1 | 1 | 10 | 21 | 12 | 9.1 | 30 |
| 17(R)-RvD1 (375/141) | 375.2 | 141.1 | 1 | 1 | 10 | 21 | 12 | 9.3 | 30 |
| RvD2 (375/175) | 375.2 | 175.1 | 1 | 1 | 10 | 30 | 12 | 8.7 | 30 |
| RvD3 (375/147) | 375.2 | 147.1 | 1 | 1 | 10 | 25 | 12 | 8.7 | 30 |
| 17(R)-RvD3 (375/147) | 375.2 | 147.1 | 1 | 1 | 10 | 25 | 12 | 8.5 | 30 |
| RvD4 (375/101) | 375.2 | 101.1 | 1 | 1 | 10 | 22 | 12 | 10 | 30 |
| RvD5 (359/199) | 359.2 | 199.1 | 1 | 1 | 10 | 21 | 12 | 11.9 | 30 |
| PD1 (359/153) | 359.2 | 153.1 | 1 | 1 | 10 | 21 | 12 | 12.1 | 30 |
| PDX (359/153) | 359.2 | 153.1 | 1 | 1 | 10 | 21 | 12 | 11.9 | 30 |
| Maresin 1 (359/221) | 359.2 | 221.1 | 1 | 1 | 10 | 20 | 12 | 12.1 | 30 |
| Maresin 1 (359/250) | 359.2 | 250.1 | 1 | 1 | 10 | 20 | 12 | 12.1 | 30 |
| Maresin 2 (359/221) | 359.2 | 221.1 | 1 | 1 | 10 | 20 | 12 | 13.1 | 30 |
| 4-HDHA (343/101) | 343.2 | 101.1 | 1 | 1 | 10 | 17 | 12 | 16.3 | 30 |
| 7-HDHA (343/141) | 343.2 | 141.1 | 1 | 1 | 10 | 18 | 12 | 15.8 | 30 |
| 13-HDHA (343/193) | 343.2 | 193.1 | 1 | 1 | 10 | 17 | 12 | 15.6 | 30 |
| 14-HDHA (343/205) | 343.2 | 205.1 | 1 | 1 | 10 | 17 | 12 | 15.7 | 30 |
| 16-HDHA (343/233) | 343.2 | 233.1 | 1 | 1 | 10 | 20 | 12 | 15.4 | 30 |
| 17-HDHA (343/245) | 343.2 | 245.1 | 1 | 1 | 10 | 17 | 12 | 15.5 | 30 |
| 7,8-EpDPA (343/113) | 343.2 | 113.1 | 1 | 1 | 10 | 20 | 12 | 16.8 | 30 |
| 10,11-EpDPA (343/153) | 343.2 | 153.2 | 1 | 1 | 10 | 20 | 12 | 16.6 | 30 |
| 13,14-EpDPA (343/161) | 343.2 | 161.1 | 1 | 1 | 10 | 20 | 12 | 16.58 | 30 |
| 16,17-EpDPA (343/274) | 343.2 | 274.1 | 1 | 1 | 10 | 20 | 12 | 16.55 | 30 |
| 19,20-EpDPA (343/241) | 343.2 | 241.1 | 1 | 1 | 10 | 20 | 12 | 16.3 | 30 |
| DHA (327/283) | 327.2 | 283.1 | 1 | 1 | 10 | 18 | 12 | 17.8 | 30 |
| DPA (329/285) | 329.2 | 285.1 | 1 | 1 | 10 | 18 | 12 | 17.9 | 30 |
| Adrenic Acid (331/287) | 331.2 | 287.1 | 1 | 1 | 10 | 18 | 12 | 18 | 30 |
| 9-HODE (295/171) | 295.2 | 171.1 | 1 | 1 | 10 | 25 | 12 | 15 | 30 |
| 13-HODE (295/195) | 295.2 | 195.1 | 1 | 1 | 10 | 25 | 12 | 15.02 | 30 |
| 13-OxoODE (13-KODE) (293/113) | 293.2 | 113.2 | 1 | 1 | 10 | 30 | 12 | 14.84 | 30 |
| 12(13)-EpOME (295/195 | 295.3 | 195.2 | 1 | 1 | 10 | 25 | 12 | 16 | 30 |
| 9,10 DiHOME (313/201) | 313.3 | 201.3 | 1 | 1 | 10 | 30 | 12 | 13 | 30 |
| 10-Nitrolinoleate (324/277) | 324.2 | 277.1 | 1 | 1 | 10 | 18 | 12 | 16.7 | 30 |
| 9(S)HOTrE (293/171) | 293.3 | 171.1 | 1 | 1 | 10 | 25 | 12 | 13.74 | 30 |
| 13(S)HOTrE (293/195) | 293.2 | 195 | 1 | 1 | 10 | 25 | 12 | 13.94 | 30 |
| PAF (508/59) | 508.3 | 59.1 | 1 | 1 | 10 | 20 | 12 | 17.8 | 30 |
|  |  |  |  |  |  |  |  |  |  |

**Supplementary Table 2. Positive Mode**

| **Compound ID** | **Q1** | **Q3** | **Edit Dwell** | **Dwell Time** | **EP** | **CE** | **CXP** | **Expected RT (min)** | **RT Tolerance (s)** |
| --- | --- | --- | --- | --- | --- | --- | --- | --- | --- |

| LTC4-d5 (631/194) | 631.3 | 194.1 | 1 | 1 | 9 | 28 | 15 | 11.5 | 30 |
| --- | --- | --- | --- | --- | --- | --- | --- | --- | --- |
| LTD4-d5 (502/194) | 502.3 | 194.1 | 1 | 1 | 9 | 28 | 13 | 10.2 | 30 |
| LTE4-d5 (445/194) | 445.2 | 194.1 | 1 | 1 | 9 | 23.5 | 13 | 12.32 | 30 |
| LTC4 (626/189) | 626.3 | 189.1 | 1 | 1 | 10 | 28 | 13 | 11.5 | 30 |
| LTD4 (497/189) | 497.3 | 189.1 | 1 | 1 | 10 | 23 | 13 | 10.22 | 30 |
| LTE4 (440/189) | 440.3 | 189.1 | 1 | 1 | 10 | 23 | 13 | 12.3 | 30 |
| MCTR1 (650/191) | 650.3 | 191.1 | 1 | 1 | 10 | 28 | 13 | 11.3 | 30 |
| MCTR2 (521/191) | 521.3 | 191.1 | 1 | 1 | 10 | 23 | 13 | 10.1 | 30 |
| MCTR3 (464/191) | 464.3 | 191.1 | 1 | 1 | 10 | 25 | 13 | 12.24 | 30 |
| PCTR1 (650/231) | 650.3 | 231.1 | 1 | 1 | 10 | 28 | 13 | 11.3 | 30 |
| PCTR2 (521/231) | 521.3 | 231.1 | 1 | 1 | 10 | 23 | 13 | 10.14 | 30 |
| PCTR3 (464/231) | 464.3 | 231.1 | 1 | 1 | 10 | 23 | 13 | 12.3 | 30 |
| PAF (524/184) | 524.3 | 183.9 | 1 | 1 | 10 | 20 | 13 | 17.8 | 30 |
| PGE2 Ethanolamide (PGE2-EA) (396/62) | 396.5 | 62.1 | 1 | 1 | 10 | 25 | 13 | 6 | 30 |
| Oleoyl Ethanolamide (OEA) (326/62) | 326.4 | 62.1 | 1 | 1 | 10 | 25 | 13 | 17.9 | 30 |
| Palmitoyl Ethanolamide (300/62) | 300.4 | 62.1 | 1 | 1 | 10 | 25 | 13 | 16.6 | 30 |
| Anandamide (AEA) (348/62) | 348.4 | 62.1 | 1 | 1 | 10 | 25 | 13 | 17.6 | 30 |
| Docosahexaenoyl Ethanolamide (DHEA) (372/62) | 372.4 | 62.1 | 1 | 1 | 10 | 25 | 13 | 17.7 | 30 |
| Linoleoyl Ethanolamide (LEA) (324/62) | 324.4 | 62.1 | 1 | 1 | 10 | 25 | 13 | 17.5 | 30 |
| Stearoyl Ethanolamide (ceramid) (328/62) | 328.4 | 62.1 | 1 | 1 | 10 | 25 | 13 | 18 | 30 |
| oxy-Arachidonoyl Ethanolamide (oxy-AEA) (364/62) | 364 | 62 | 1 | 1 | 10 | 25 | 13 | 14.5 | 30 |
| 2-Arachidonoyl Glycerol (2AG) (379/287) | 379.4 | 287.2 | 1 | 1 | 10 | 25 | 13 | 17.73 | 30 |
| Docosatetraenoyl Ethanolamide (DEA) (376/62) | 376.6 | 62.1 | 1 | 1 | 10 | 25 | 13 | 17.9 | 30 |
| alpha-linolenoyl ethanolamide (322/62) | 322.4 | 62.1 | 1 | 1 | 10 | 25 | 13 | 16.4 | 30 |
| oleamide (282/247) | 282.4 | 247.4 | 1 | 1 | 10 | 25 | 13 | 17.9 | 30 |
| dihomo-gamma-linolenoyl ethanolamide (350/62) | 350.4 | 62.1 | 1 | 1 | 10 | 25 | 13 | 17.8 | 30 |
| docosanoyl ethanolamide (384/62) | 384.5 | 62.1 | 1 | 1 | 10 | 25 | 13 | 19 | 30 |

**Supplementary Table 3. Percentage of eicosanoids detected in female synovial fluid at 2 weeks post-DMM**

| **Sample #_Strain** | 1_*FD^-/-^* | 2_*FD^-/-^* | 3_*FD^-/-^* | 4_*FD^-/-^* | 5_WT | 6_WT | **7_WT** | **8_WT** |
| --- | --- | --- | --- | --- | --- | --- | --- | --- |
| Not Detected | 8 | 11 | 11 | 8 | 9 | 13 | **4*** | **4*** |
| Detected (out of 30) | 22 | 19 | 19 | 22 | 21 | 17 | **26*** | **26*** |
| Percentage | 73% | 63% | 63% | 73% | 70% | 57% | **87%*** | **87%*** |

**Supplementary Table 4. Correlations between pressure-pain threshold and normalized abundances of eicosanoids of interest in the synovial fluid and serum**

| **Synovial Fluid – Data points are combined across all sex, strains, and time points** | | | | | |  |  |  |  |
| --- | --- | --- | --- | --- | --- | --- | --- | --- | --- |
|  | **12-HHT** | **Palmitoyl Ethanolamide** | **EPA** | **Arachidonic Acid** | **15-HETE** | | **12-HETE** | **13-HODE** | **14-HDHA** |
| Spearman's coefficient | ***-0.39**** | -0.02 | 0.05 | -0.07 | -0.01 | | -0.17 | -0.21 | ***0.38**** |
| p-value | ***0.03**** | ns | ns | ns | ns | | ns | ns | ***0.03**** |
| **Serum – Data points are combined across all sex, strains, and time points** | | | | |  | |  |  |  |
|  | **12-HHT** | **Palmitoyl Ethanoloamide** | **EPA** | **Arachidonic Acid** | **15-HETE** | | **12-HETE** | **13-HODE** | **14-HDHA** |
| Spearman's coefficient | 0.12 | -0.01 | 0.01 | 0.16 | -0.08 | | 0.12 | -0.15 | 0.07 |
| p-value | ns | ns | ns | ns | ns | | ns | ns | ns |

**** = p ≤ 0.05 correlation***

**Supplementary Table 5. Normalized abundances of eicosanoids of interest between male and female mice**

|  | **WT** | | **FDKO** | |
| --- | --- | --- | --- | --- |
| **2 weeks Post-DMM: Serum** | **Male** | **Female** | **Male** | **Female** |
| 12,HHT | ***5.61**** | ***5.41**** | 5.41 | 5.4 |
| Palmitoyl Ethanolamide | ***5.54**** | ***5.93**** | ***5.49**** | ***5.78**** |
| EPA | 7.22 | 7.14 | 7.22 | 7.33 |
| Arachidonic Acid | 6.91 | 7.35 | ***6.98**** | ***7.27**** |
| **2 weeks Post-DMM: Synovial Fluid** | **Male** | **Female** | **Male** | **Female** |
| 12-HETE | 5.26 | 5.85 | ***5.72**** | ***5.3**** |
| 13-HODE | ***5.70**** | ***5.62**** | ***5.9**** | ***5.63**** |
| 14-HDHA | ***2.95**** | ***4.42**** | 3.08 | 2.75 |
| **8 weeks Post-DMM: Serum** | **Male** | **Female** | **Male** | **Female** |
| 14-HDHA | ***5.88**** | ***5.71**** | 5.76 | 5.71 |
| 15-HETE | 5.18 | 5.06 | 4.88 | 5.04 |
| **** = p ≤ 0.05 within genotype*** |  |  |  |  |


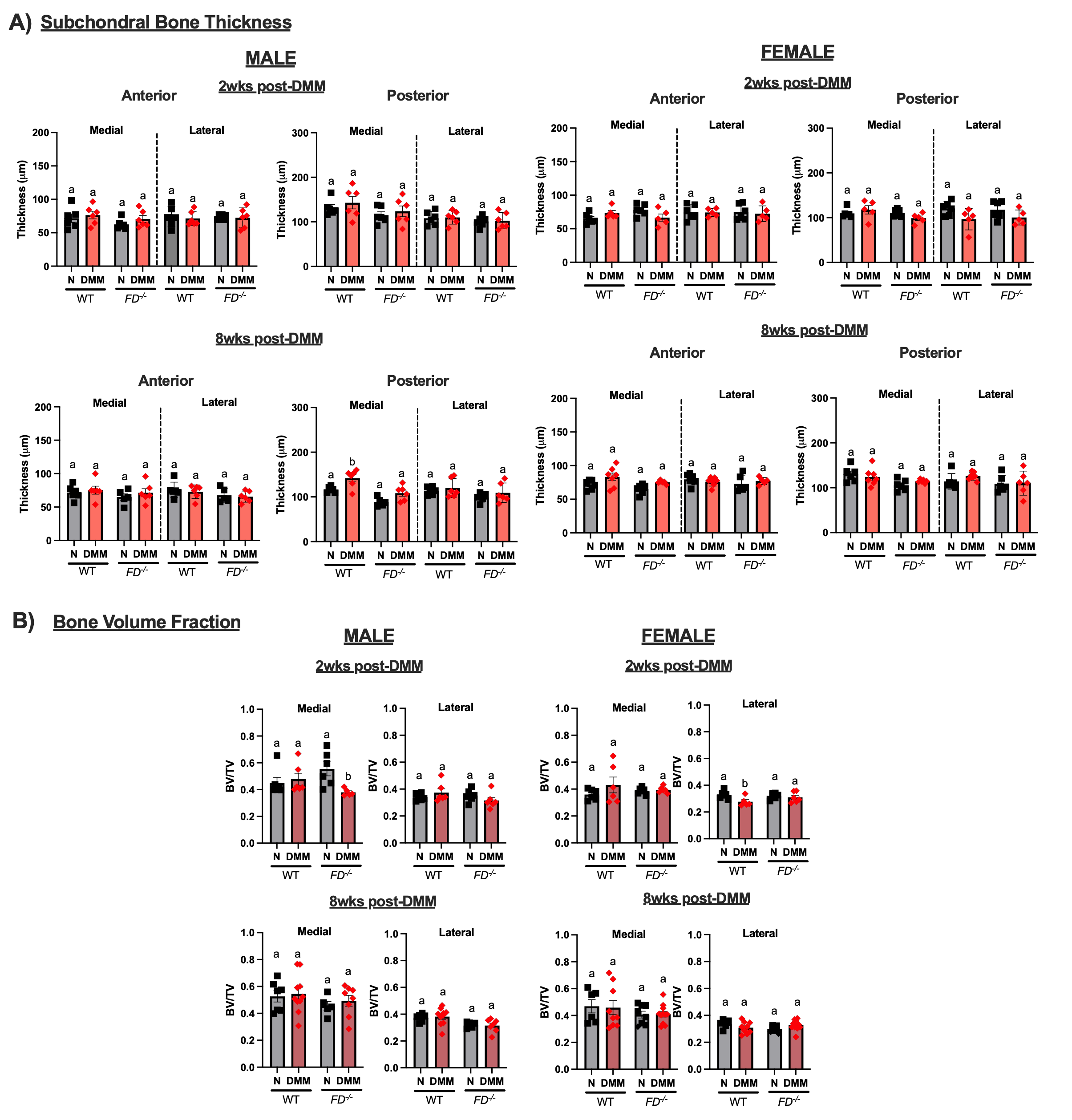


**Supplementary Figure 1. There were changes in the subchondral bone microarchitecture as a result of DMM** (A) Subchondral bone thickness of the distal femur was greater in the posterior medial compartment in WT male mice 8 weeks post-DMM. There were no differences in subchondral bone thickness at 2 weeks post-DMM in male mice. There were no differences in female due to surgery or strain. B) Bone volume fraction (BV/TV) was significantly lower in the medial compartment of the proximal tibial epiphysis in male *FD^-/-^* mice and in the lateral compartment in female WT mice 2 weeks post-DMM. There were no other significant differences between strain, surgery, or sex in the proximal tibial epiphysis. Two-way ANOVA with Sidak’s post-hoc test was used to analyze between surgery within each strain. Different letters represent p<0.05 when comparing naïve and DMM groups within each strain. N = naïve, n=6-11 per surgery/sex/strain.

**
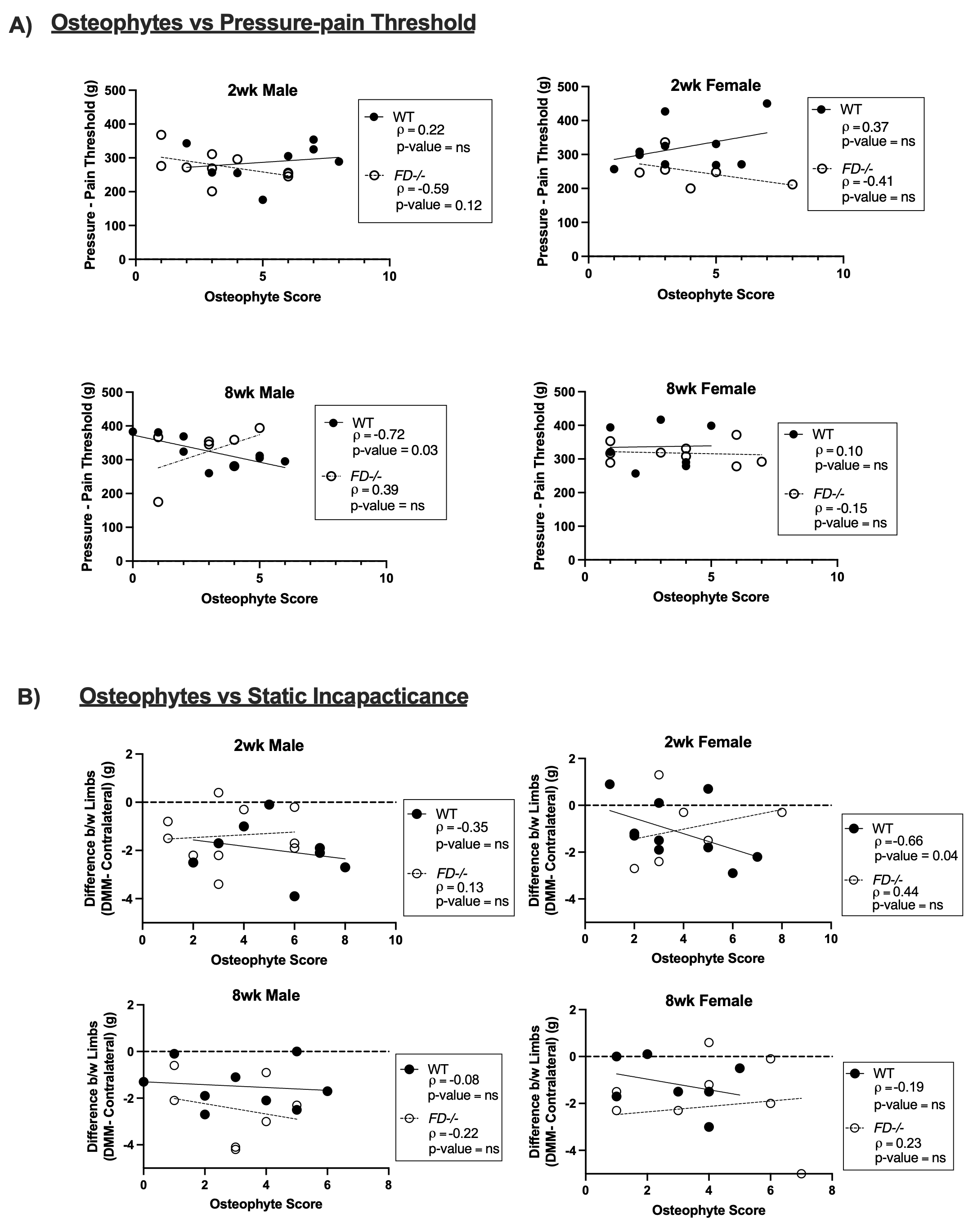
**

**Supplementary Figure 2. Correlations between osteophyte scores with pressure-pain threshold or static incapacitance within sexes.** (A,B) *FD^-/-^* mice exhibited no significant Spearman’s correlation between osteophytes and pressure-pain threshold or osteophytes and offloading of the surgical limb in either sex at 2 or 8 weeks-post DMM. Spearman’s correlation coefficient (ρ) is reported. p<0.05 is indicated in the graphs. ns = not significant, n=6-11 per sex/strain.


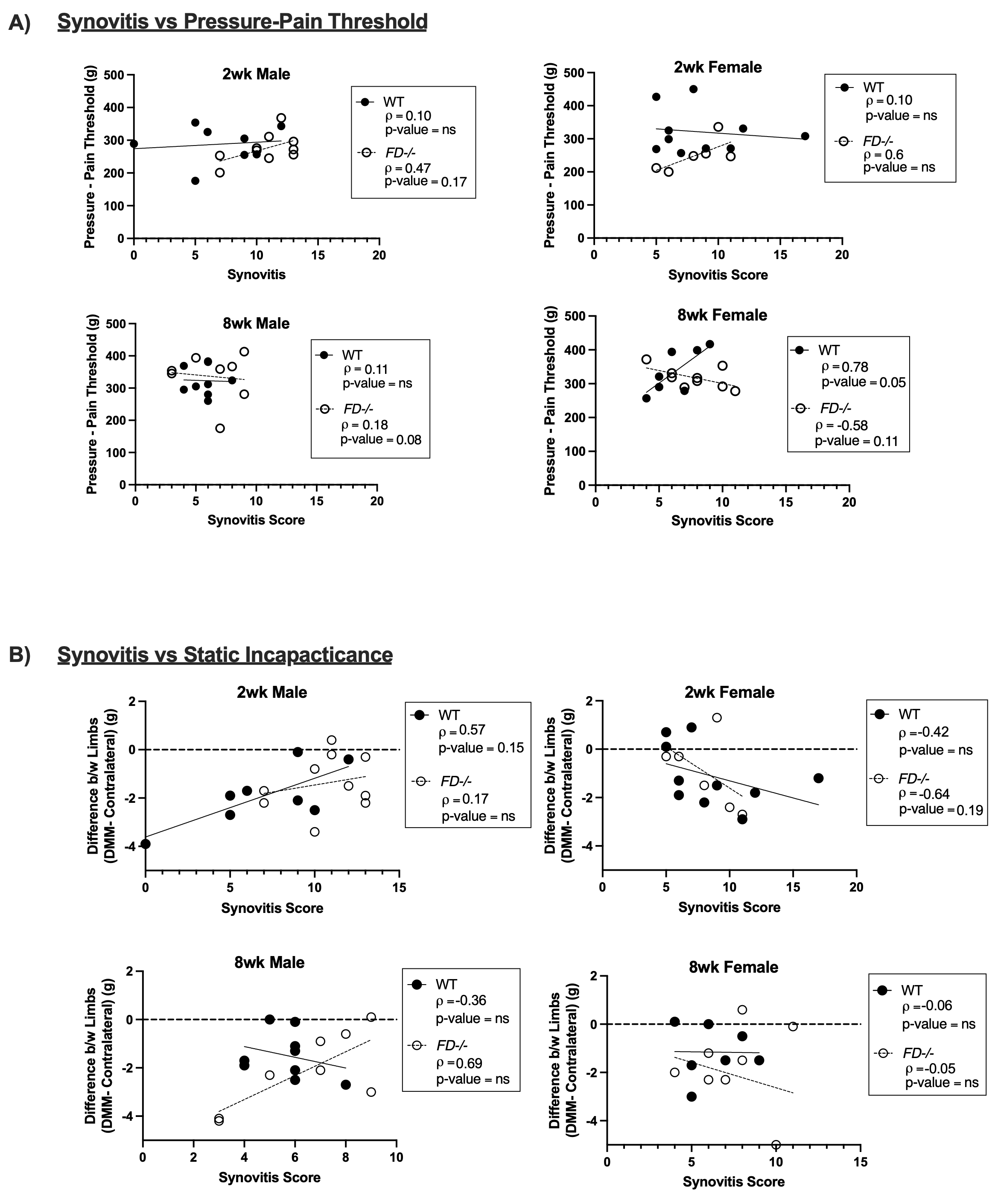


**Supplementary Figure 3. Correlations between synovitis scores with pressure-pain threshold or static incapacitance within sexes.** (A,B) *FD^-/-^* mice exhibited no significant Spearman’s correlation between osteophytes and pressure-pain threshold or osteophytes and offloading of the surgical limb in either sex at 2 or 8 weeks-post DMM. Spearman’s correlation coefficient (ρ) is reported. p<0.05 is indicated in the graphs. ns = not significant, n=6-11 per sex/strain.


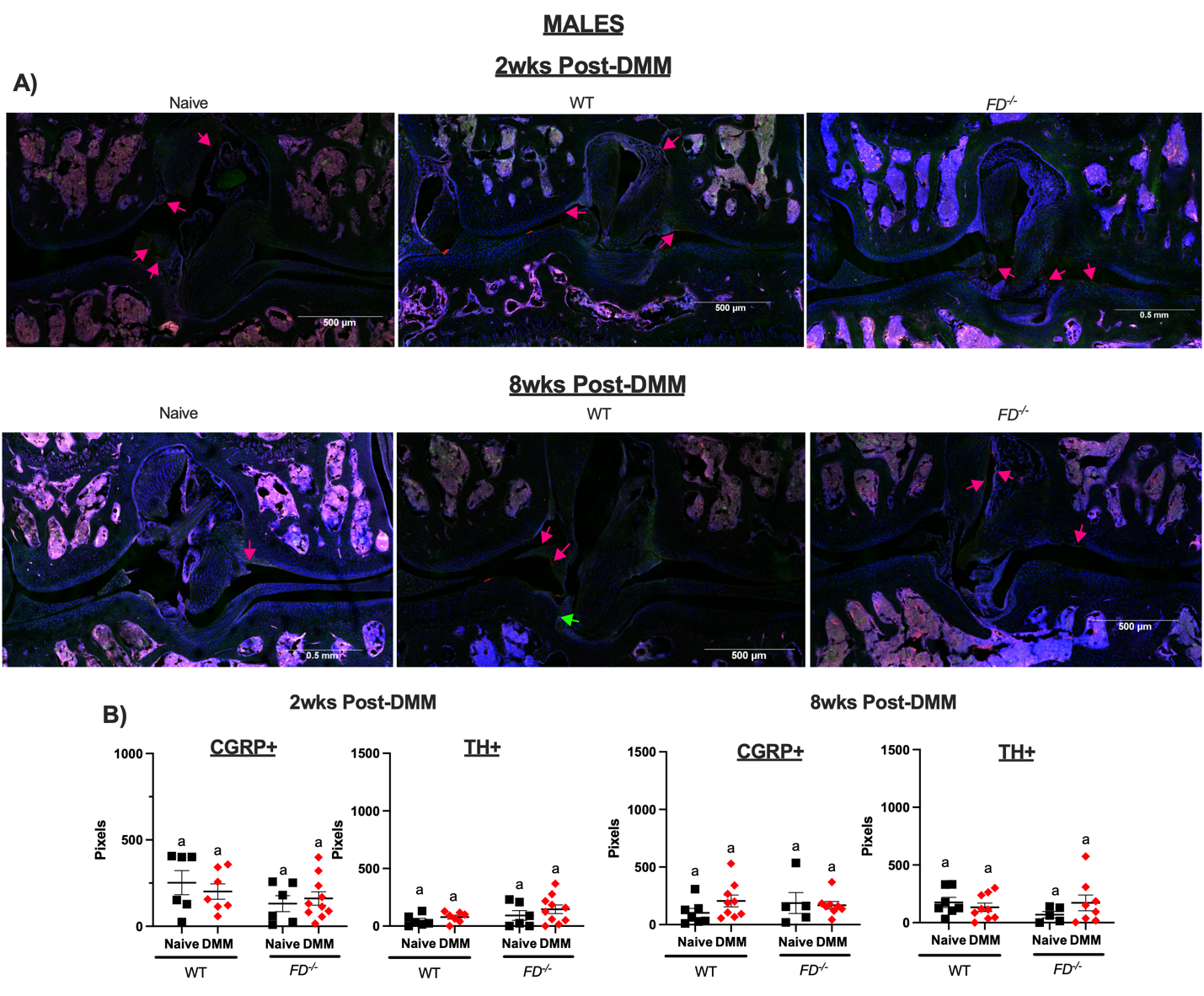


**Supplementary Figure 4. There were no detectable differences in number of CGRP+ and TH+ endings in male mice.** (A) 10x representative images of male joints at 2 and 8 weeks post-DMM stained for CGRP (red arrows) and TH (green arrows) (B) Preliminary analysis of CGRP+ and TH+ showed no clear differences between surgery or strain in male mice. Two-way ANOVA with Sidak’s post-hoc test was used to analyze between surgery within each strain. Different letters represent p<0.05 when comparing naïve and DMM groups within each strain, n=5-10 per surgery/strain.

**
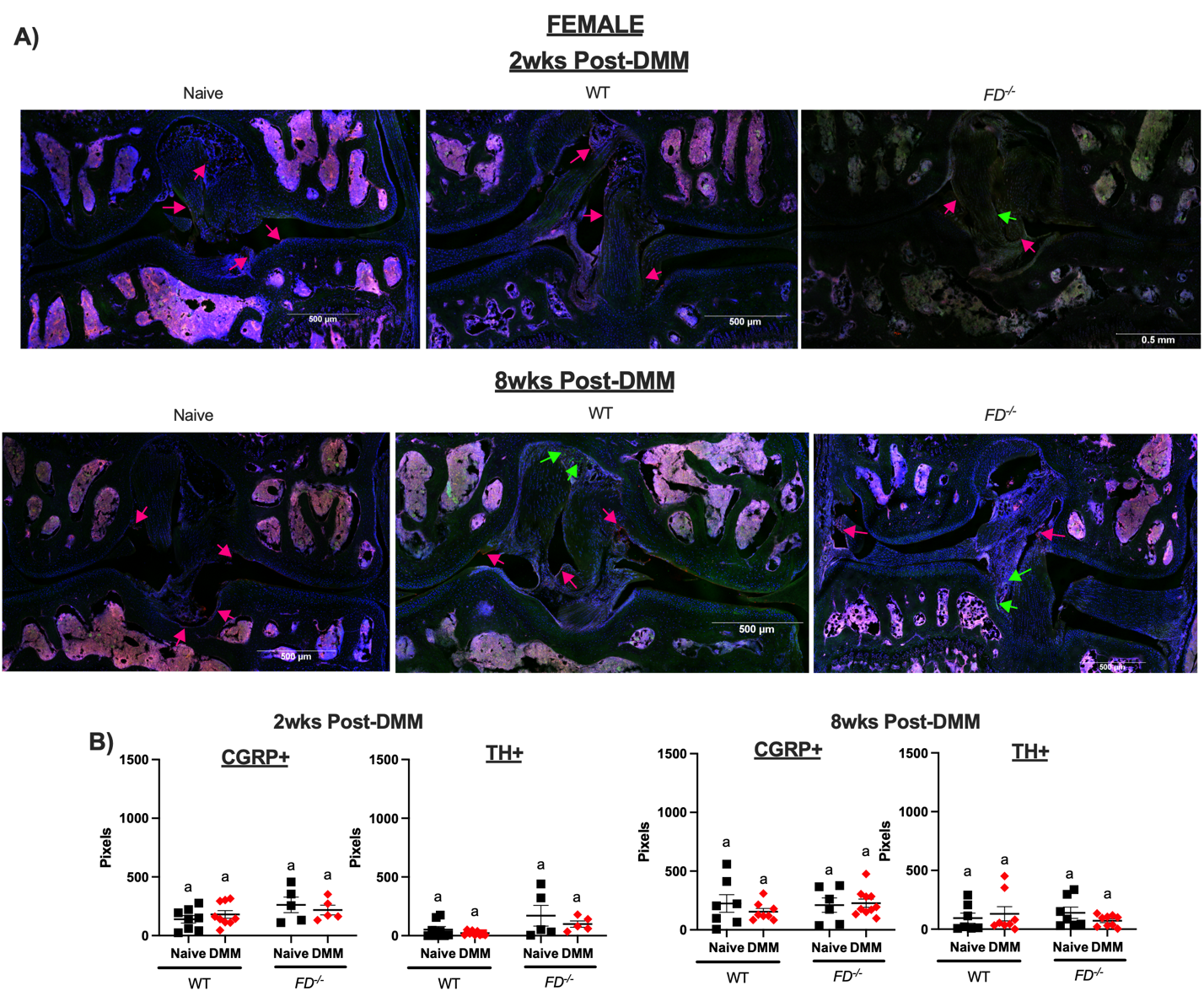
Supplementary Figure 5. There were no detectable differences in number of CGRP+ and TH+ endings in female mice.** (A) 10x representative images of female joints at 2 and 8 weeks post-DMM stained for CGRP (red arrows) and TH (green arrows) (A) Preliminary analysis of CGRP+ and TH+ neurites showed no statistical differences in between strain or surgery at the joint. Two-way ANOVA with Sidak’s post-hoc test was used to analyze between surgery within each strain. Different letters represent p<0.05 when comparing naïve and DMM groups within each strain, n=5-10 per surgery/strain.


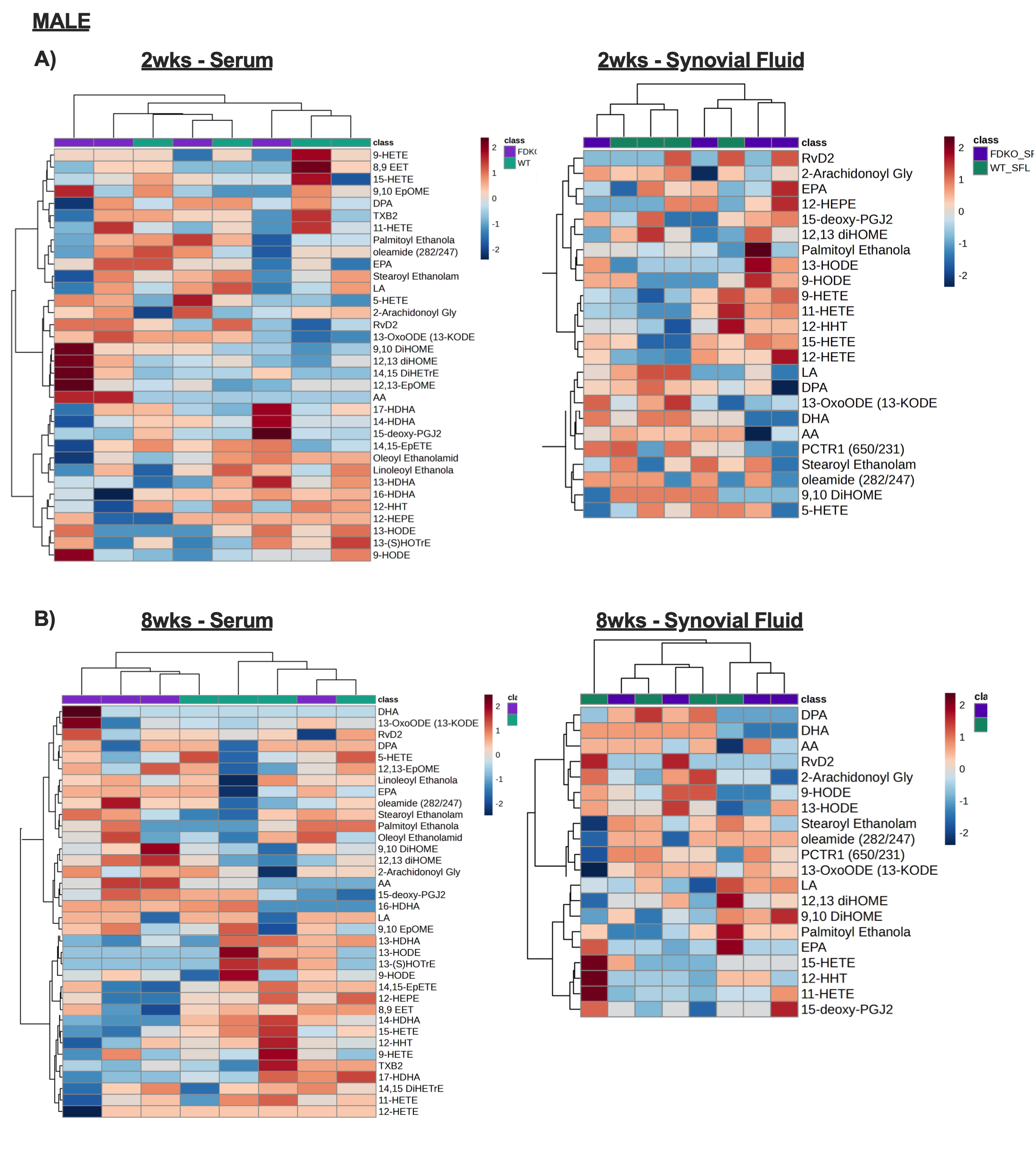
**Supplementary Figure 6. Heat maps of targeted lipidomic profiles of male *FD^-/-^* and WT DMM groups.** (A) 2 weeks and (B) 8 weeks post-DMM in male mice.

**
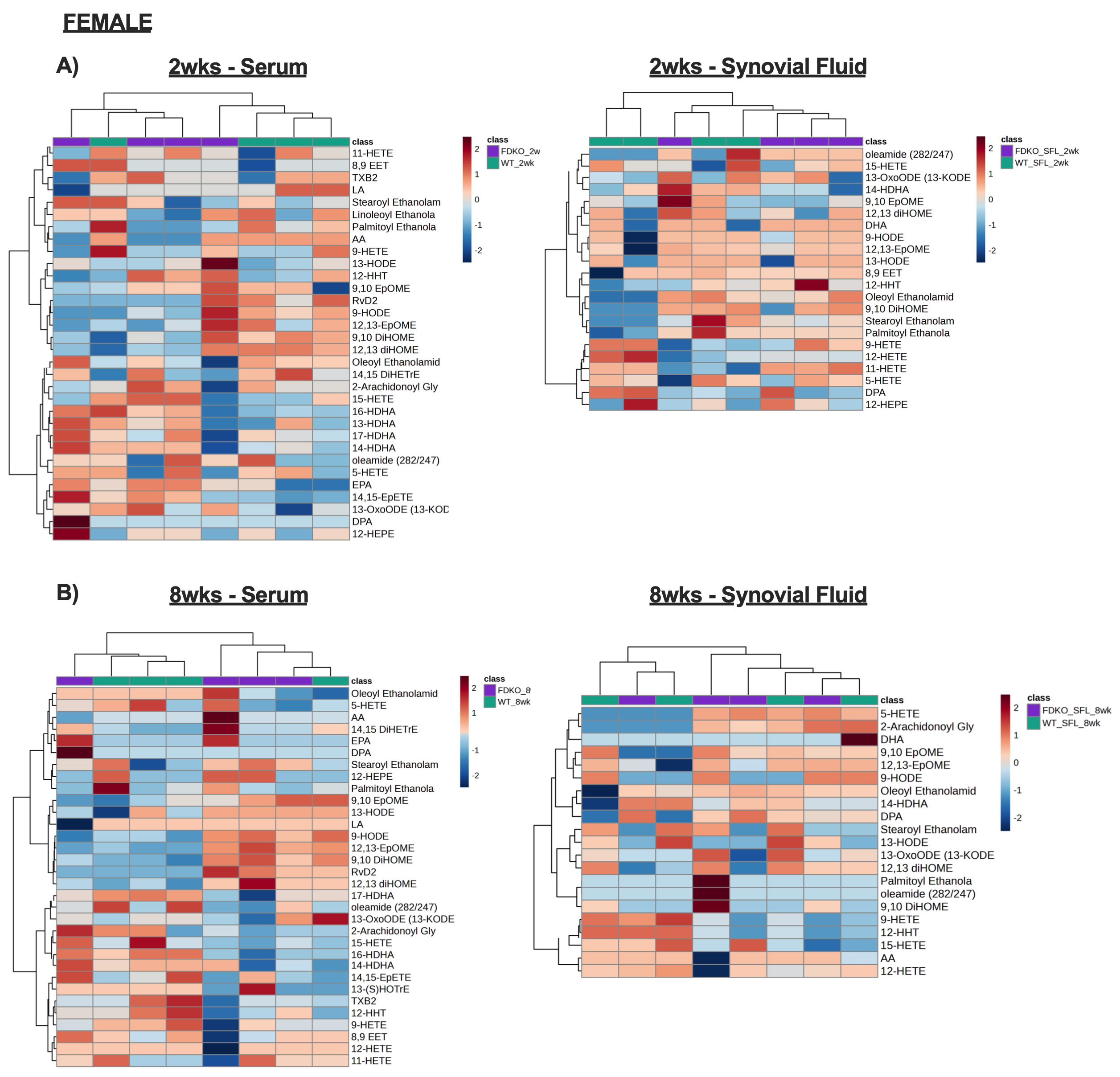
Supplementary Figure 7. Heat maps of targeted lipidomic profiles of female *FD^-/-^* and WT DMM groups.** (A) 2 weeks and (B) 8 weeks post-DMM in female mice.

**
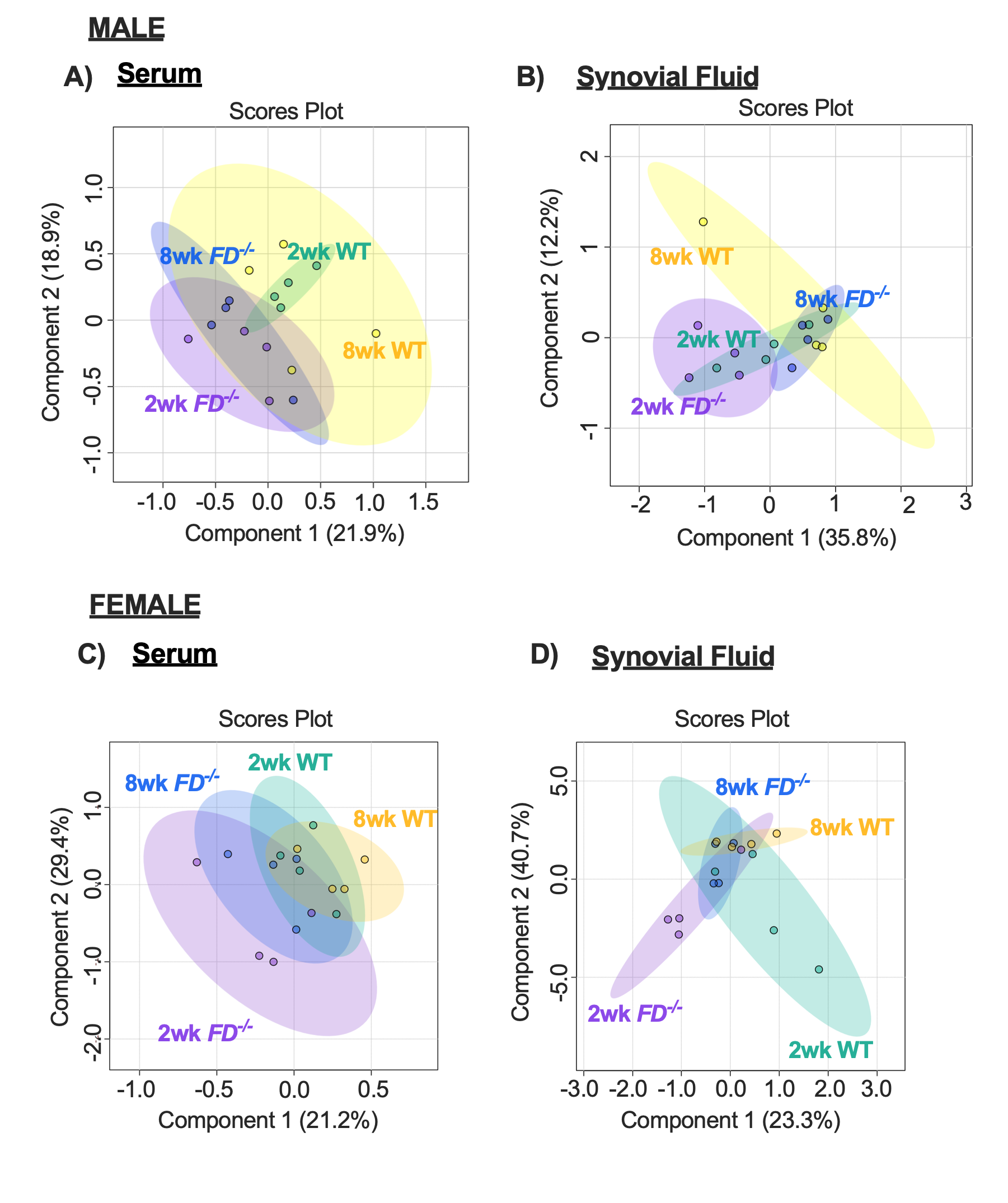
Supplementary Figure 8. PLS-DA plot comparing strain and timepoint separated into four groups.** (A) Serum (B) Synovial fluid of male mice. (C) Serum (D) Synovial fluid of female mice.
